# Schistosomiasis endemicity and its role in sexually transmitted infections – a systematic review and meta-analysis

**DOI:** 10.3389/fpara.2024.1451149

**Published:** 2024-09-02

**Authors:** Kwame Kumi Asare, Philip Afful, Godwin Kwami Abotsi, Czarina Owusua Adu-Gyamfi, George Benyem, Gnatoulma Katawa, Kathrin Arndts, Manuel Ritter

**Affiliations:** ^1^ Biomedical and Clinical Research Centre, College of Health and Allied Sciences, University of Cape Coast, Cape Coast, Ghana; ^2^ Department of Biomedical Sciences, School of Allied Health Sciences, College of Health and Allied Sciences, University of Cape Coast, Cape Coast, Ghana; ^3^ Department of Immunology, Noguchi Memorial Institute for Medical Research, University of Ghana, Accra, Ghana; ^4^ Unité de Recherche en Immunologie et Immunomodulation (UR2IM)/Laboratoire de Microbiologie et de Contrôle de Qualité des Denrées Alimentaires (LAMICODA), Ecole Supérieure des Techniques Biologiques et Alimentaires, Université de Lomé, Lomé, Togo; ^5^ Institute for Medical Microbiology, Immunology, and Parasitology (IMMIP), University Hospital Bonn (UKB), Bonn, Germany; ^6^ German-West African Centre for Global Health and Pandemic Prevention (G-WAC), partner site Bonn, Bonn, Germany

**Keywords:** female genital schistosomiasis (FGS), sexually transmitted infections (STIs), schistosomiasis-endemic communities, curable STIs, meta-analysis, public health interventions

## Abstract

**Introduction:**

Schistosomiasis, a tropical parasitic disease, affects 779 million people globally, with 85% of cases in Africa. The interplay between schistosomiasis and other sexually transmitted infections (STIs) can exacerbate health burdens, but most attention has focused on interactions with HIV, neglecting coinfections with other STIs. This systematic review and meta-analysis aims to understand the role *Schistosoma* infections play in STIs within schistosomiasis-endemic populations.

**Methods:**

The study is a systematic review and meta-analysis investigating the link between Schistosoma infections and STIs in endemic regions. It uses PRISMA guidelines, electronic databases, and Google Scholar to assess prevalence, associations, and heterogeneity, reducing bias using a Meta-Mar statistical tool.

**Results:**

A quantitative synthesis of 33 articles from 1975–2024 involved 22,587 participants from 13 countries, including regions in Africa, France, and China, examining coinfections of schistosomiasis and STIs, including HIV. The pooled estimates showed a significant risk association between schistosomiasis and STIs [RR (95% CI) = 1.18, (1.13–1.24); z/t = 7.55, p<0.0001] using a fixed effect model. Cochran’s Q test (Tau^2^ = 0.5061, Chi^2^ = 476.65, df = 32, p<0.01) indicated significant heterogeneity. The Higgins I^2^ statistic of 93.0% (91.5%–94.7%), H = 3.86 (3.43–4.33), highlighted substantial variance between studies. Subgroup analysis showed West Africa [Weight IV = 1.7%, RR (95% CI) = 1.78 (1.28–2.47), I^2^ = 59%], East Africa [Weight IV = 10.5%, RR (95% CI) = 0.99 (0.86–1.13), I^2^ = 54%], and Southern Africa [Weight IV = 82.0%, RR (95% CI) = 1.16 (1.10–1.21), I^2^ = 97%] contributed significantly to the high heterogeneity in the pooled analysis. Females had a notably higher risk of STIs in the context of schistosomiasis (k = 17, RR: 1.30, 95% CI: 1.23–1.37, Q = 316.78, I^2^ = 94.9%), compared to males (k = 6, RR: 0.94, 95% CI: 0.77–1.15, Q = 53.44, I^2^ = 90.6%) and the combined group of females and males (k = 9, RR: 0.95, 95% CI: 0.88–1.02, Q = 16.38, I^2^ = 50.2%).

**Conclusion:**

The study found a high risk of coinfections between schistosomiasis and STIs, particularly in West and Southern Africa, confirming female genital schistosomiasis as a major risk for STIs.

## Introduction

Parasitic infections, caused by protozoa, helminths, and ectoparasites, significantly impact human health by causing substantial morbidity, chronic illness, and disability, particularly in tropical and subtropical regions, while co-infections with diseases like HIV complicate management (Oluwafemi et al., 2022; Ngui et al., 2024). The zoonotic potential of these infections, such as toxoplasmosis, highlights the importance of controlling animal infections to prevent transmission to humans, necessitating integrated control strategies including mass drug administration and improved sanitation, with a particular focus on diseases like schistosomiasis, which is associated with several pathogenic infections that devastate affected individuals and complicate disease management (Fernández Romero, 2015; Casillas-Vega et al., 2017; Aggarwal et al., 2022; Ayejoto et al., 2023).

Schistosomiasis, a neglected tropical parasitic disease, affects 779 million people globally, with 85% in Africa. 207 million people in 74 countries are infected, with 120 million developing symptoms (World Health Organization, 2013; World Health Organization, 2017). Africa bears the brunt of the burden, housing 90% of infected populations requiring treatment, emphasizing the need for targeted public health interventions (World Health Organization, 2021).

Sexually transmitted infections (STIs) are caused by over 30 bacteria, viruses, and parasites, with eight pathogens leading the highest incidence (Lewis, 2011; Deshmukh et al., 2023). Both curable STIs include syphilis, gonorrhea, chlamydia, and trichomoniasis, and incurable viral infections such as herpes simplex virus (HSV), human immunodeficiency virus (HIV), and human papillomavirus (HPV) require attention, especially in regions with helminth coinfections (Deshmukh et al., 2023; Elendu et al., 2024). STIs have a significant impact on sexual and reproductive health worldwide, with more than 1 million new STIs acquired every day (Smolarczyk et al., 2021; Elendu et al., 2024). In 2020, the World Health Organization (WHO) estimated 374 million new infections with one of four major STIs: chlamydia (129 million), gonorrhea (82 million), syphilis (7.1 million), and trichomoniasis (156 million) (Shrier, 2005). Additionally, more than 490 million people were living with genital herpes in 2016, and an estimated 300 million women have an HPV infection, which is the primary cause of cervical cancer and anal cancer among men who have sex with men (Shrier, 2005; Bustinduy et al., 2022).

In Africa, there are 69 million new treatable STIs each year (Yirenya-Tawiah et al., 2014). Similarly, low- and middle-income countries bear a disproportionately high burden of STIs such as HSV, HIV, and HPV (Gwitira et al., 2018; Power, 2019; Anthonj et al., 2022). STIs have a profound impact on women’s health, causing conditions such as cervicitis, urethritis, and pelvic inflammatory disease, which can lead to serious reproductive health complications and poor pregnancy outcomes (Rollinson, 2009; Mawa et al., 2021).

STIs are highly prevalent in areas endemic to schistosomiasis (Kjetland et al., 2005; Jordens, 2019). The co-occurrence of these infections is often driven by overlapping risk factors, including poor access to healthcare, a lack of education, and socio-economic challenges (Kjetland et al., 2014; Livingston et al., 2021). In such regions, the interplay between schistosomiasis and STIs can exacerbate the health burden on affected populations, complicating diagnosis, treatment, and prevention efforts (Van Tong et al., 2017; Parums, 2021). Several studies have reported coinfection between schistosomiasis and STIs such as syphilis, gonorrhea, chlamydia, trichomoniasis, HSV, HIV, and HPV (Migliavaca et al., 2020; Nakagawa et al., 2022; Putri et al., 2023; Topçuoğlu and Arsava, 2023). Despite this, most attention has been focused on the interaction between schistosomiasis and HIV, often neglecting the significant impact of coinfections with other STIs (Nakagawa et al., 2022; Putri et al., 2023). The female genital schistosomiasis (FGS), is classified as a Group 1 biological carcinogen by the International Agency for Research on Cancer (IARC) due to its significant association with cervical cancer (Mayaud et al., 1992).

Addressing this gap in coinfections involving schistosomiasis and a wider range of STIs is essential for improving health outcomes. By expanding the focus to include syphilis, gonorrhea, chlamydia, trichomoniasis, HSV, HPV, and other STIs, alongside HIV, public health initiatives can better address the complexities of these coinfections. A One Health approach, which integrates human, animal, and environmental health efforts, along with ongoing research and international collaboration, is crucial for effectively addressing and combating parasitological challenges (Fernández Romero, 2015; Casillas-Vega et al., 2017). This inclusive approach can enhance diagnostic strategies, treatment protocols, and preventive measures, ultimately leading to more effective management of these interconnected health challenges.

This systematic review and meta-analysis focused on understanding the role schistosoma infections play in STIs in the schistosomiasis endemic population. The outcome of the study could offer insight into integrated health strategies to enhance diagnostic strategies, treatment protocols, and preventive measures that address both schistosomiasis and STIs to improve overall health outcomes and reduce the disease burden in these communities.

## Materials and methods

### Literature search strategy

The literature search was conducted using the guide and protocol provided by the Preferred Reporting Items for Systematic Reviews and Meta-Analyses (PRISMA) with the PRISMA checklist (**Supplementary Table S1**) (Ansart et al., 2005). The literature search was conducted from the electronic databases of Scopus, PubMed, Medline, Science Direct, Cochrane, and ClinicalTrails.gov to identify peer-reviewed articles about schistosomiasis or female genital schistosomiasis and sexually transmitted infections published between January 1, 1975, and May 1, 2024 (**Supplementary Table S2**). A manual search of articles relevant to the study was conducted using the Google Scholar search engine. The boolean operators “OR” and “AND” were used to create the search query, and the search was limited to full articles that were open access and published in the English language within a year of publication and relevant to the study area. The bibliographies of the selected articles were manually compiled, cleaned, categorized, and assessed for citations.

### Study eligibility criteria

The study screened articles related to schistosomiasis and sexually transmitted infections, the prevalence of schistosomiasis among people with STIs and/or HIV, and the association of HIV or other sexually transmitted infections with schistosomiasis. This study compiled and analyzed original research articles focused on human participants from diverse demographics and geographic regions. The inclusion and exclusion criteria were designed to ensure the selection of relevant articles that contribute to a comprehensive understanding of the topic. The inclusion criteria typically involve original research articles involving human participants of any age, gender, race, or geographical location. The focus was on studies that directly investigate the prevalence of schistosomiasis among individuals with STIs or HIV, as well as the association between these conditions. Exclusion criteria encompass experimental studies involving non-human subjects, review articles, letters to editors, duplicated studies, and articles lacking relevant keywords or deemed irrelevant to the study’s aims. To ensure that data duplication is addressed when incorporating studies from ClinicalTrials.gov into published articles, a thorough and systematic approach is followed. First, all identified articles are loaded into EndNote referencing software version 20.2.1. This software automatically detects and removes duplicate articles, streamlining the process of managing references. Next, to further ensure that ClinicalTrials.gov data is not duplicated in the analysis, a search for clinical trial registration numbers is performed on the remaining eligible articles. This step involves cross-referencing the trial registry numbers (e.g., NCT numbers) with those listed in the articles. By comparing these unique identifiers, any remaining instances of duplicated data are identified and resolved. This rigorous approach ensures that the final dataset is free from duplication, enhancing the reliability and accuracy of the systematic review or meta-analysis. The combination of automated duplicate detection and manual verification of trial registry numbers provides a robust method for maintaining data integrity and ensuring that the findings accurately reflect the available evidence.

### Study selection and data extraction

A systematic and rigorous approach to data collection and analysis was demonstrated after searching and combining the selected articles by searching multiple databases, removing duplicates, and comprehensively capturing relevant literature on the topic. The screening process, conducted by two independent reviewers, ensured that articles met the predefined inclusion criteria for further analysis. During the full-text review, data extraction focused on various aspects, including publication details, methodology, study setting, population characteristics, diagnostic tests employed, as well as key results such as identified schistosomiasis and STIs. This comprehensive approach aimed to gather a broad range of information relevant to the study’s objectives. Importantly, the inclusion of two independent reviewers helped enhance the study’s reliability by reducing the likelihood of bias in the selection and extraction of data. Additionally, the consensus reached through discussion, particularly when a third reviewer had differing opinions, further strengthened the study’s validity and reliability. The overall methodological approach underscores the commitment to rigor and thoroughness in gathering and analyzing data related to the association between schistosomiasis and STIs and/or HIV.

### Assessment of study quality and risk of bias

The Joanna Briggs Institute critical appraisal checklist was used to assess the quality of the studies (Kjetland et al., 2008). The process involves applying nine criteria, with each rated as “YES” or “NO”. A scoring system was then used to assign a numerical value to each study based on the number of criteria met. Studies were categorized as low quality (scores 0–4), moderate quality (scores 5–7), or high quality (scores 8–9). The study included studies rated as moderate to high quality (**Supplementary Table S3**) based on the Joanna Briggs Institute critical appraisal checklist guidelines for Quality assessment (**Supplementary Table S4**). Two independent reviewers assessed the quality, reducing bias and enhancing reliability. Consensus discussions were used to resolve discrepancies. This standardized method ensures rigorous study inclusion.

### Data analysis

The study utilized a rigorous statistical approach to analyze data from multiple sources regarding the prevalence of schistosomiasis, particularly in association with STIs and/or HIV. All data were entered into Microsoft Excel (WA, USA) and statistically analyzed using Meta-Mar v3.5.1 (https://meta-mar.shinyapps.io/meta-analysis-calculator/) (Downs et al., 2013; Hegertun et al., 2013), employed for descriptive statistical tests (Dichotomous models; risks and ratios and average effect size using log risk ratio) and meta-analysis, respectively. A fixed-effects and random-effects models, incorporating log transformation and the restricted maximum likelihood method, were chosen to calculate pooled datasets, considering the expected between-study and within-study variances in a large meta-analysis. Subgroup analyses were conducted based on the geographical area where the study was conducted. Heterogeneity among studies was assessed through visual inspection of forest plots, Cochran’s Q test, and Higgins’ inconsistency statistic (I^2^), with an I^2^ value above 50% indicating substantial heterogeneity. This meticulous approach ensured a robust analysis of schistosomiasis prevalence and its association with STIs and HIV.

### Publication bias

The study also addressed publication bias and heterogeneity to ensure the robustness of its findings. Publication bias was evaluated through Fail-safe N calculation using the Rosenthal Approach, funnel plots, and Egger’s test to assess the possibility of selecting the publication of studies based on their results (Galappaththi-Arachchige et al., 2016). Higgins’ I^2^ statistic was utilized to quantify the degree of heterogeneity between studies, with high heterogeneity observed across factors such as the geographical area where the study was conducted. Sensitivity analyses were conducted to examine the influence of the largest studies on the meta-analyses, allowing for a comprehensive assessment of the data’s reliability and consistency. These approaches provided a thorough evaluation of potential biases and variations in the data, strengthening the validity and credibility of the study’s conclusions regarding the association between schistosomiasis, STIs, and HIV.

## Results

### Study characteristics

**Figure 1:**
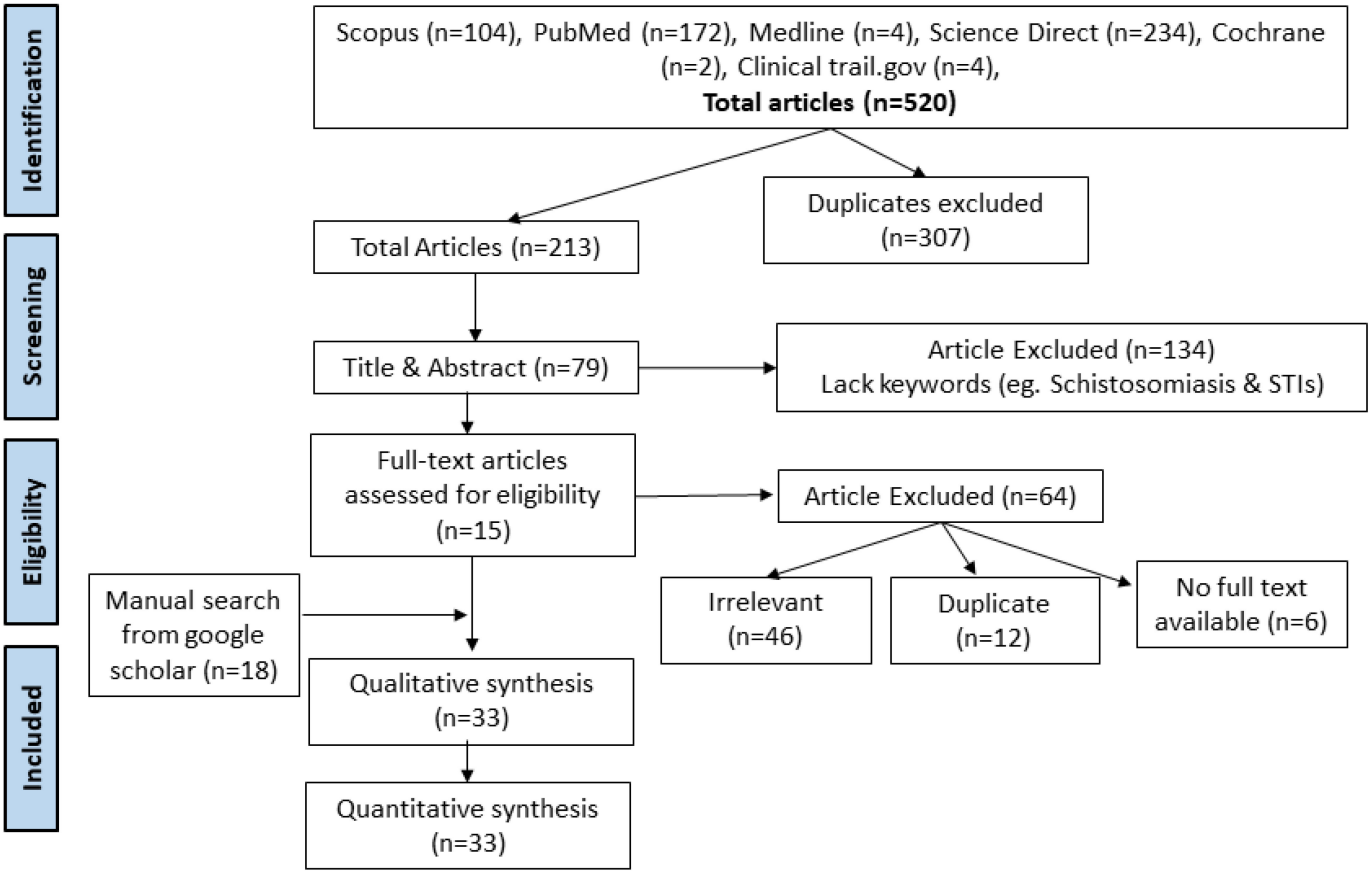
PRISMA flow chart for search and selection of included studies.

A systematic review of 520 studies was conducted using six electronic databases: Scopus (n = 104), PubMed (n = 172), Medline (n = 4), Science Direct (n = 234), Cochrane (n = 2), ClinicalTrails.gov (n = 4). After removing duplicates and screening titles and abstracts, 15 full-text articles assessed qualified for eligibility for inclusion. A manual search for “schistosomiasis and sexually transmitted infections” from the Google Scholar database yielded an additional 18 articles. Thus, a total of 33 articles were used for quantitative synthesis (**Figure 1**) (McCarthy et al., 1989; Fontanet et al., 2000; Watson-Jones et al., 2000; Wilson et al., 2001; Leutscher et al., 2003; Mosunjac et al., 2003; Kallestrup et al., 2005; Leutscher et al., 2005; Kjetland et al., 2006; Ndhlovu et al., 2007; Kjetland et al., 2010; Downs et al., 2011; Downs et al., 2012; Yirenya-Tawiah et al., 2013; Mazigo et al., 2014; Njoku, 2014; Prodger et al., 2015; Sanya et al., 2015; Christinet et al., 2016; Midzi et al., 2017; Downs et al., 2017a; Downs et al., 2017b; Galappaththi-Arachchige et al., 2018; Wall et al., 2018; Yang et al., 2018; Yegorov et al., 2018; Colombe et al., 2018a; Gadoth et al., 2019; Sturt et al., 2020; Patel et al., 2021; Sturt et al., 2021; Kutz et al., 2023; Shukla et al., 2023). The database search retrieved published articles that were written in English with full open access and published between the years 1975 and 2024. In all, 22,587 participants were involved in the 33 studies from 13 countries, 11 from Africa, 1 from France (European migrants), and 1 from China that reported coinfections of schistosomiasis and STIs, including HIV, were used for the quantitative meta-analysis (**Table 1**). The most common STIs with their prevalences across the various studies included in the quantitative analysis were *Neisseria gonorrhoeae* (McCarthy et al., 1989; Leutscher et al., 2003; Ndhlovu et al., 2007; Njoku, 2014; Galappaththi-Arachchige et al., 2018), *Chlamydia trachomatis* (McCarthy et al., 1989; Leutscher et al., 2003; Ndhlovu et al., 2007; Njoku, 2014; Downs et al., 2017b; Galappaththi-Arachchige et al., 2018; Gadoth et al., 2019; Shukla et al., 2023), HIV (Fontanet et al., 2000; Watson-Jones et al., 2000; Wilson et al., 2001; Mosunjac et al., 2003; Kallestrup et al., 2005; Leutscher et al., 2005; Kjetland et al., 2006; Ndhlovu et al., 2007; Kjetland et al., 2010; Downs et al., 2012; Yirenya-Tawiah et al., 2013; Mazigo et al., 2014; Njoku, 2014; Prodger et al., 2015; Sanya et al., 2015; Midzi et al., 2017; Downs et al., 2017a; Wall et al., 2018; Yang et al., 2018; Colombe et al., 2018a; Gadoth et al., 2019; Sturt et al., 2020; Sturt et al., 2021; Kutz et al., 2023; Shukla et al., 2023), HSV-2 (Leutscher et al., 2003; Gadoth et al., 2019; Patel et al., 2021), *Treponema pallidum* (Ndhlovu et al., 2007; Downs et al., 2011; Sanya et al., 2015; Downs et al., 2017b; Wall et al., 2018; Gadoth et al., 2019), HPV (Christinet et al., 2016; Downs et al., 2017a; Yang et al., 2018; Gadoth et al., 2019), *Trichomonas vaginalis* (McCarthy et al., 1989; Njoku, 2014; Sturt et al., 2020; Shukla et al., 2023), *Candida albicans* (Sturt et al., 2020), Bacterial vaginosis (Ndhlovu et al., 2007; Downs et al., 2017b; Wall et al., 2018), *Mycoplasma genitalium* (Njoku, 2014), *L. crispatus* (Prodger et al., 2015), *L. iners* (Prodger et al., 2015), *G. vaginalis* (Prodger et al., 2015), and *A. vaginae* (Prodger et al., 2015). The frequent diagnostic methods employed include leucocyte esterase (LE) dipstick, enzyme-linked immunosorbent assay (ELISA), enzyme immunoassay (EIA), photo-colposcopic examination, Papanicolaou (Pap) smears, wet mounts, Kato-Katz, biopsies, serologic tests (antigen/antibody test), seroconversion, culture, gynecological examinations, polymerase chain reaction (PCR), rapid test, and schistosome Circulating Anodic Antigen (CAA) (**Supplementary Table S5**).

**Table 1:** Demographic characteristics of the 33 eligible studies included in the quantitative meta-analysis.

Study	Country	STD	N
Mayaud et al., 1992	Tanzania	*Neisseria gonorrhoeae* *Chlamydia trachomatis*	248
Ansart et al., 2005	France	STD	622
Kjetland et al., 2008	Zimbabwe	STD	483
Hegertun et al., 2013	South Africa	STD	1,057
Downs et al., 2013	Tanzanian	STD	39
Galappaththi-Arachchige et al., 2016	South Africa	STD	883
Galappaththi-Arachchige et al., 2018	South Africa	STD	1413
Yegorov et al., 2018	Uganda	STD	58
Gadoth et al., 2019	Democratic Republic of Congo	STD	367
Kjetland et al., 2006	Zimbabwe	STD	445
Downs et al., 2011	Tanzania	STD	457
Downs et al., 2017b	Tanzania	STD	207
Shukla et al., 2023	South Africa	STD	933
Leutscher et al., 2003	Madagascar	Urethritis	438
McCarthy et al., 1989	Sudan	STD	771
Downs et al., 2012	Tanzania	STD	345
Wall et al., 2018	Zambia	STD	2,145
Colombe et al., 2018a	Tanzania	STD	172
Downs et al., 2017a	Tanzania	STD, male	674
Sanya et al., 2015	Ugandan	STD	1,412
Fontanet et al., 2000	Ethiopia	STD	1,239
Ndhlovu et al., 2007	Zimbabwe	STD	544
Midzi et al., 2017	Zimbabwe	STD	1,584
Mazigo et al., 2014	Tanzania	STD	1,785
Kjetland et al., 2010	Zimbabwe	HPV/STD	236
Leutscher et al., 2005	Madagascar	STD	240
Sturt et al., 2021	Zambia	STD	410
Yirenya-Tawiah et al., 2013	Ghana	STD	402
Kallestrup et al., 2005	Zimbabwe	STD	1,545
Kutz et al., 2023	Madagascar	HPV	302
Yang et al., 2018	Southwestern China	HIV	90
Njoku, 2014	Nigeria	Hiv	1,007
Prodger et al., 2015	Ugandan	HSV-2	34

### Schistosomiasis endemicity and its role in STIs

**Figure 2:**
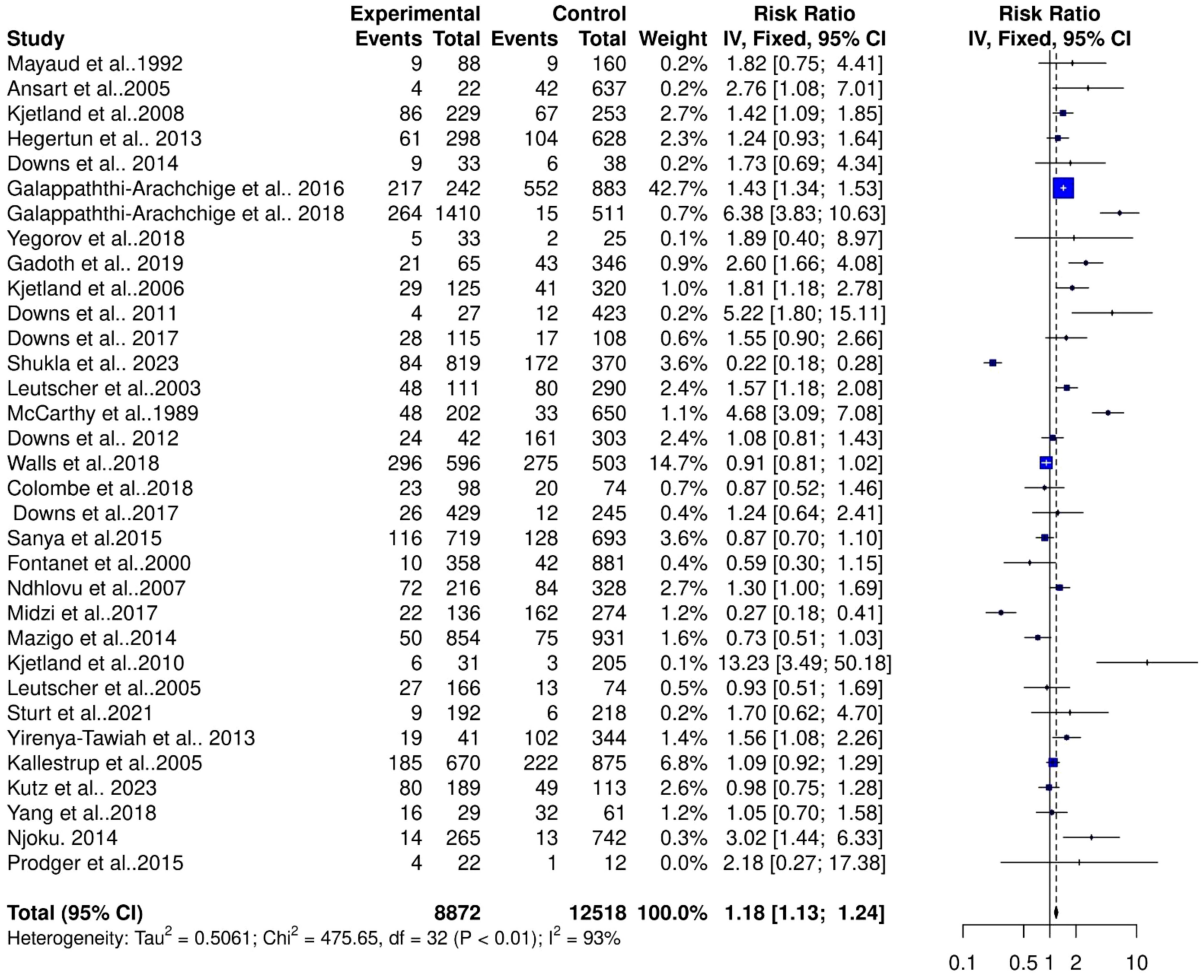
Forest plot showing fixed effect model of human schistosomiasis and sexually transmitted infection collected from 33 studies.

**Figure 3:**
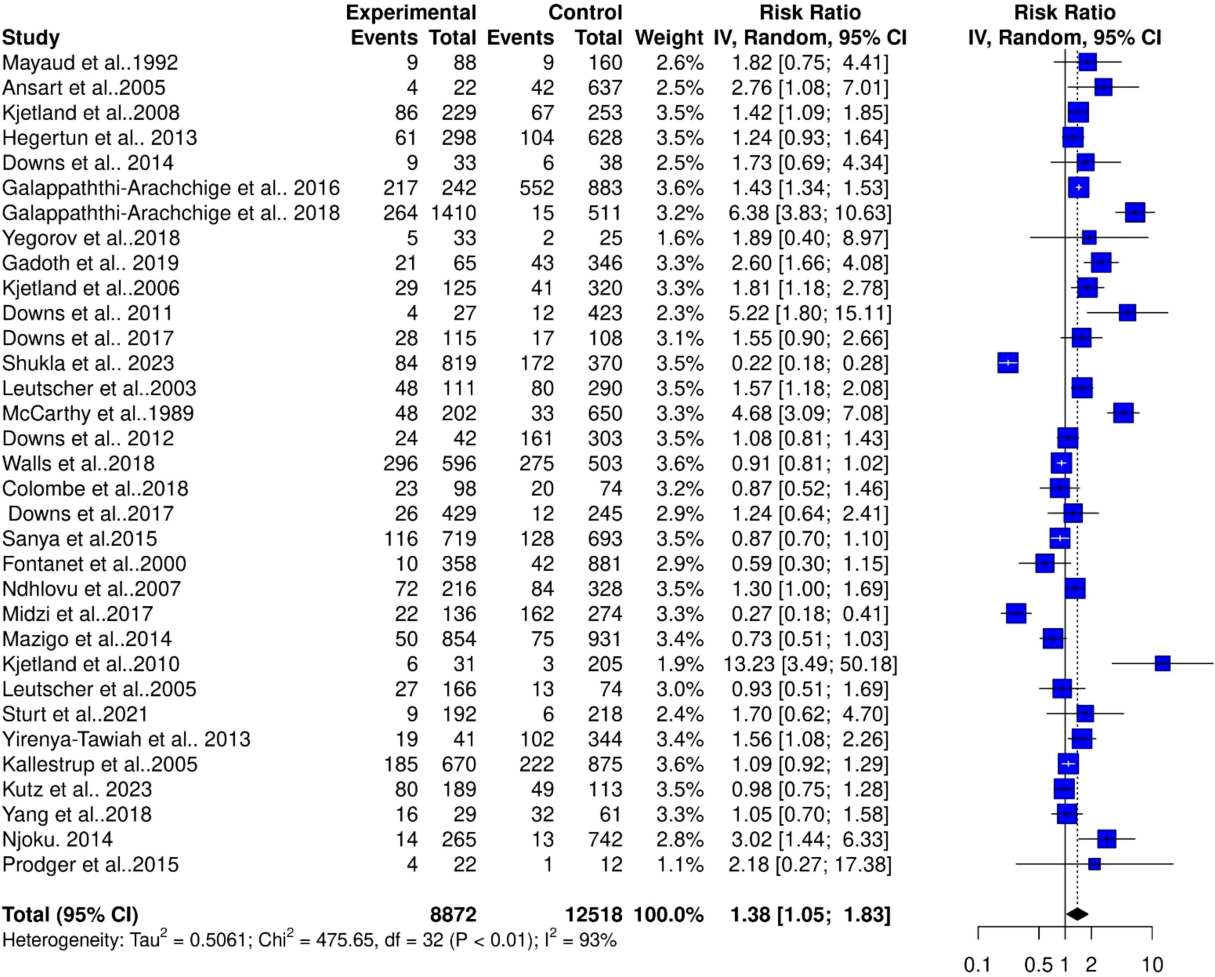
Forest plot showing random effect model of human schistosomiasis and sexually transmitted infection collected from 33 studies.

The pooled estimates from 33 studies showed a significant risk of association between schistosomiasis and STIs (RR = 1.18, 95% CI, 1.13–1.24; z/t = 7.55, p<0.0001) for the fixed effect model (**Figure 2**). The random effect model showed a slightly higher risk ratio for the association of schistosomiasis and STIs (RR = 1.38, 95% CI, 1.05–1.83; z/t = 2.39, p = 0.023) (**Figure 3**). The Cochran’s Q (chi-square) test (Tau^2^ = 0.5061, Chi^2^ = 476.65, df = 32, p<0.01) indicated the presence of significant heterogeneity. The degree of between-studies variances was substantially high, as indicated by the Higgins I^2^ (95%CI) statistic of 93.0% (91.5%–94.7%), H = 3.86 (3.43–4.33). The presence of high heterogeneity (Q = 475.65, df = 32, p<0.0001) suggests a variation in the pooled study datasets.

### Contribution of geographical area to the level of heterogeneity in the schistosomiasis endemicity and its role in STIs

**Figure 4:**
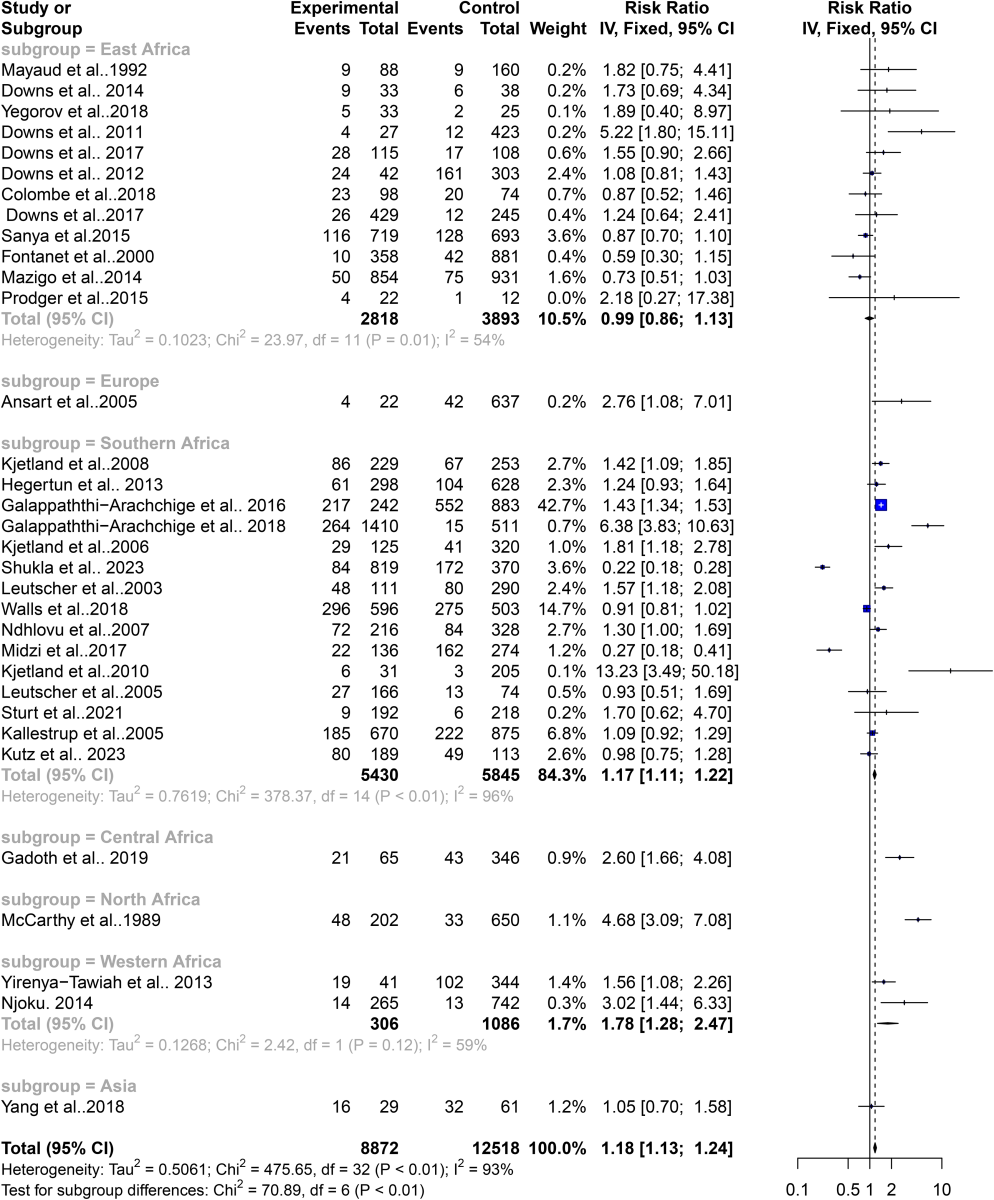
Forest plot showing fixed effect model of human schistosomiasis and sexually transmitted infection based on geographical area.

**Figure 5:**
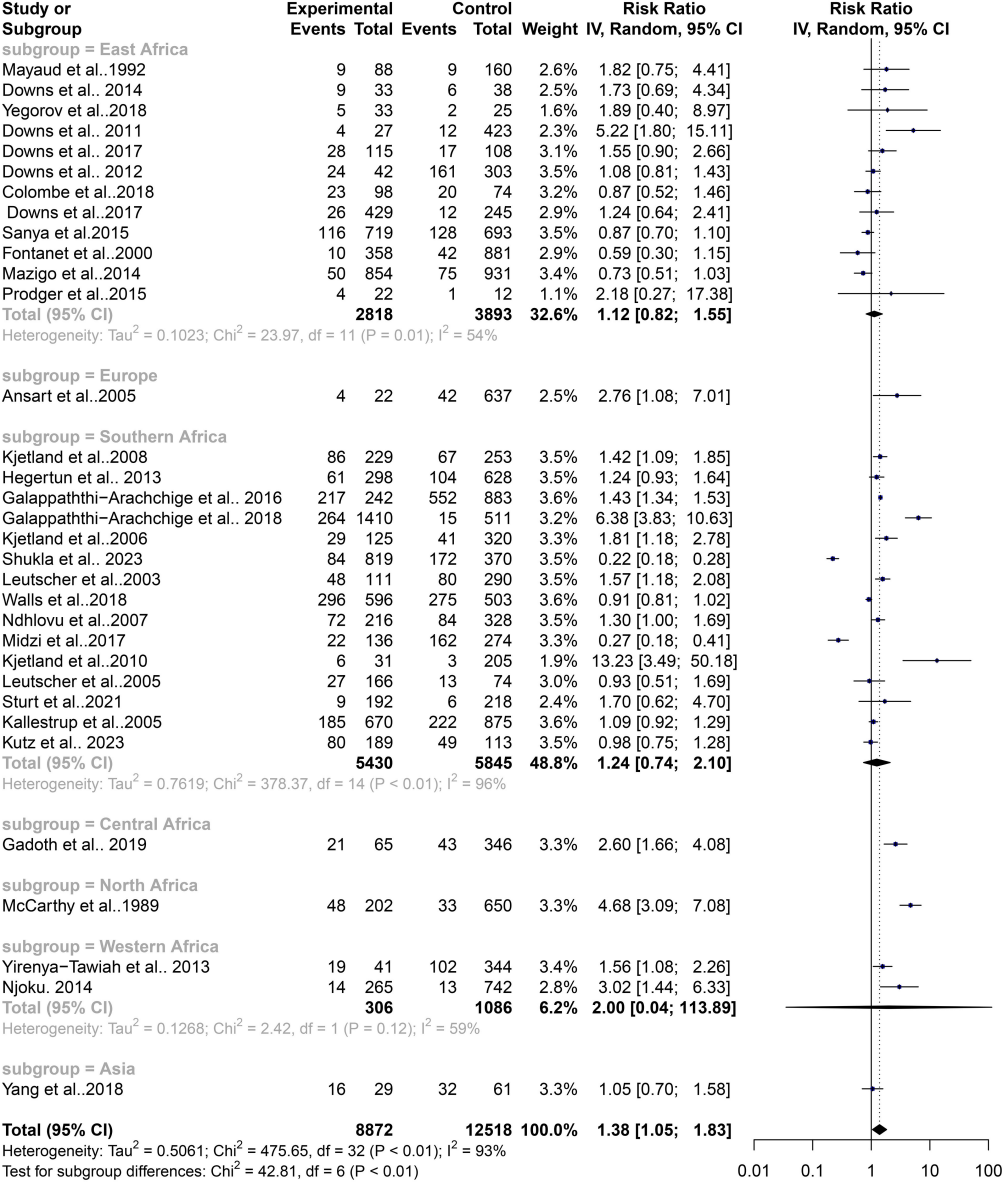
Forest plot showing random effect model of human schistosomiasis and sexually transmitted infection based on geographical area.

The high heterogeneity in the pooled estimate for datasets extracted for the analysis of schistosomiasis endemicity and its role in STIs indicates a degree of variance between studies (Cochran’s Q = 475.65, df = 32, p<0.0001), with the number of studies (k) = 33, observations (o) = 21,390, and the number of events (e) = 4,511. For the common (fixed) effect model, RR (95% CI) = 1.1826 (1.1323–1.2353), z/t = 7.55, p<0.0001 (**Figure 2**), and the random effects model, RR (95% CI) = 1.3844 (1.0491–1.8269), z/t = 2.39, p = 0.023. The pooled heterogeneity showed Tau^2^ = 0.5061 (0.299–0.9894), Tau = 0.7114 (0.5468–0.9947), I^2^ = 93.3% (91.5%–94.7%), H = 3.86 (3.43–4.33) (**Figure 4**). Thus, the geographical areas where the individual studies were conducted were a possible factor contributing to the significantly high heterogeneity among the pooled datasets. The datasets were subgrouped into seven (7) based on where studies were conducted to assess the association between schistosomiasis and STIs; Asia (1 study), Central Africa (1 study), Europe (1 study), North Africa (1 study), West Africa (2 studies), East Africa (12 studies), and Southern Africa (14 studies) were analyzed to assess their contributions to the high heterogeneity in the pooled analysis. The common effect model for subgroups showed heterogeneity between groups (Q = 75.18, df = 7, p<0.0001) and within-group heterogeneity (Q = 400.47, df = 25, p<0.0001), whereas the random effect model for the subgroup heterogeneity between groups was (Q = 42.91, df = 7, p<0.0001). The subgroups analysis of geographical areas showed studies from West Africa [Weight IV = 1.7%, RR (95% CI) = 1.78 (1.28–2.47), I^2^ = 59%], and East Africa [Weight IV = 10.5%, RR (95% CI) = 0.99 (0.86–1.13), I^2^ = 54%] and Southern Africa [Weight IV = 82.0%, RR (95% CI) = 1.16 (1.10–1.21), I^2^ = 97%] contributed the high heterogeneity in the pooled analysis by the fixed effect model (**Figure 4**). Similarly, the subgroup analysis by the random effect model for the geographical areas showed studies from West Africa [Weight IV = 6.2%, RR (95% CI) = 2.00 (0.04–113.89), I^2^ = 59%], East Africa [Weight IV = 32.6%, RR (95% CI) = 1.12 (0.82–1.55), I^2^ = 54%], and Southern Africa [Weight IV = 45.3%, RR (95% CI) = 1.23 (0.70–2.17), I^2^ = 97%] contributed to the high heterogeneity in the pooled analysis (**Figure 5**). These results suggest that individuals from West and Southern Africa had a higher risk of coinfections between schistosomiasis and STIs compared to inhabitants from East Africa.

### Contribution of gender to the level of heterogeneity in the schistosomiasis endemicity and its role in STIs

**Figure 6:**
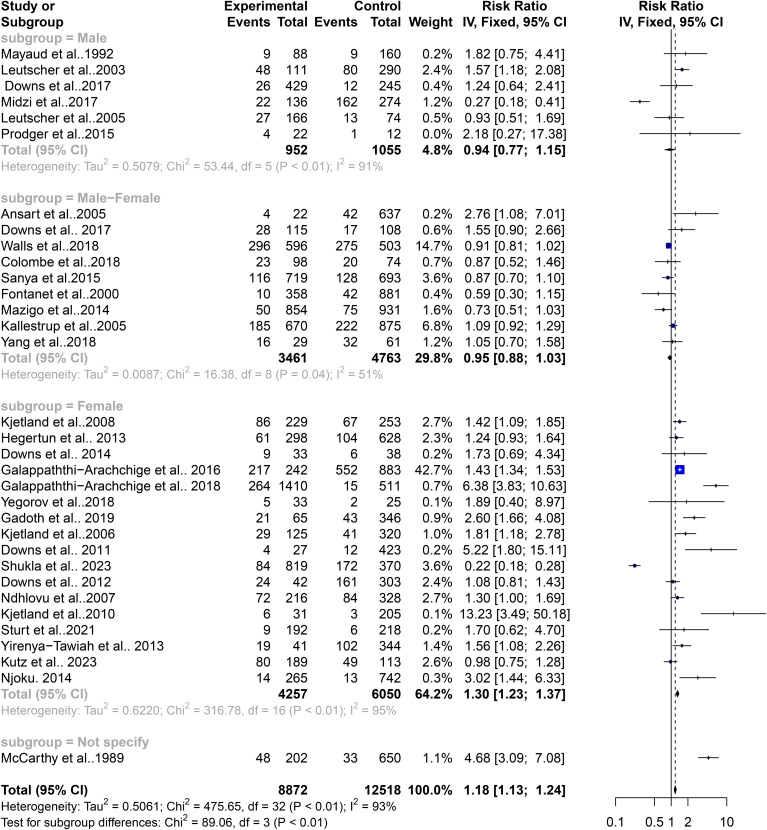
Forest plot showing fixed effect model of human schistosomiasis and sexually transmitted infection based on gender.

**Figure 7:**
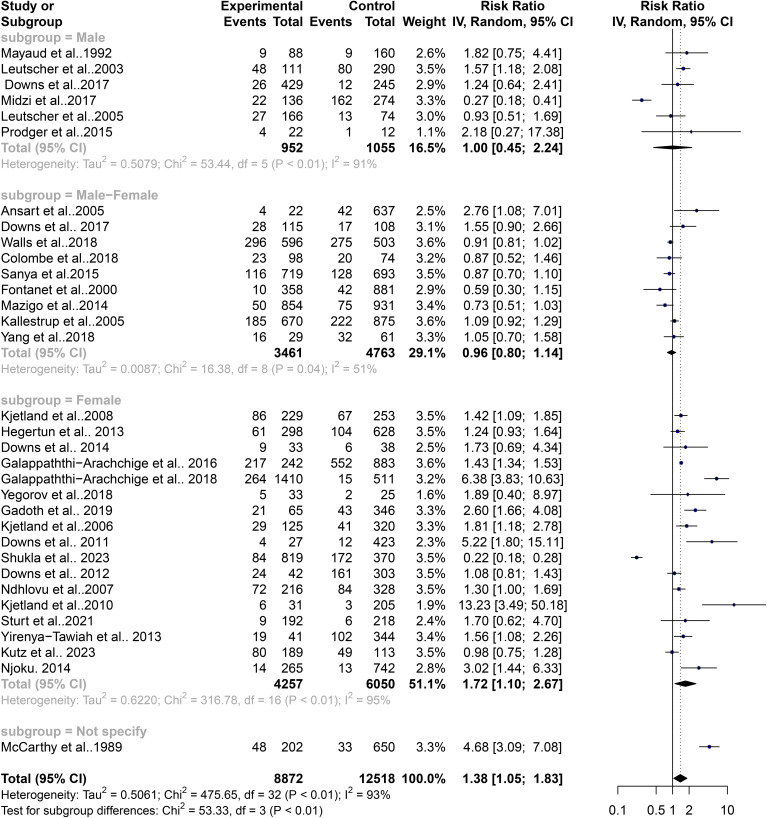
Forest plot showing random effect model of human schistosomiasis and sexually transmitted infection based on gender.

**Figure 8:**
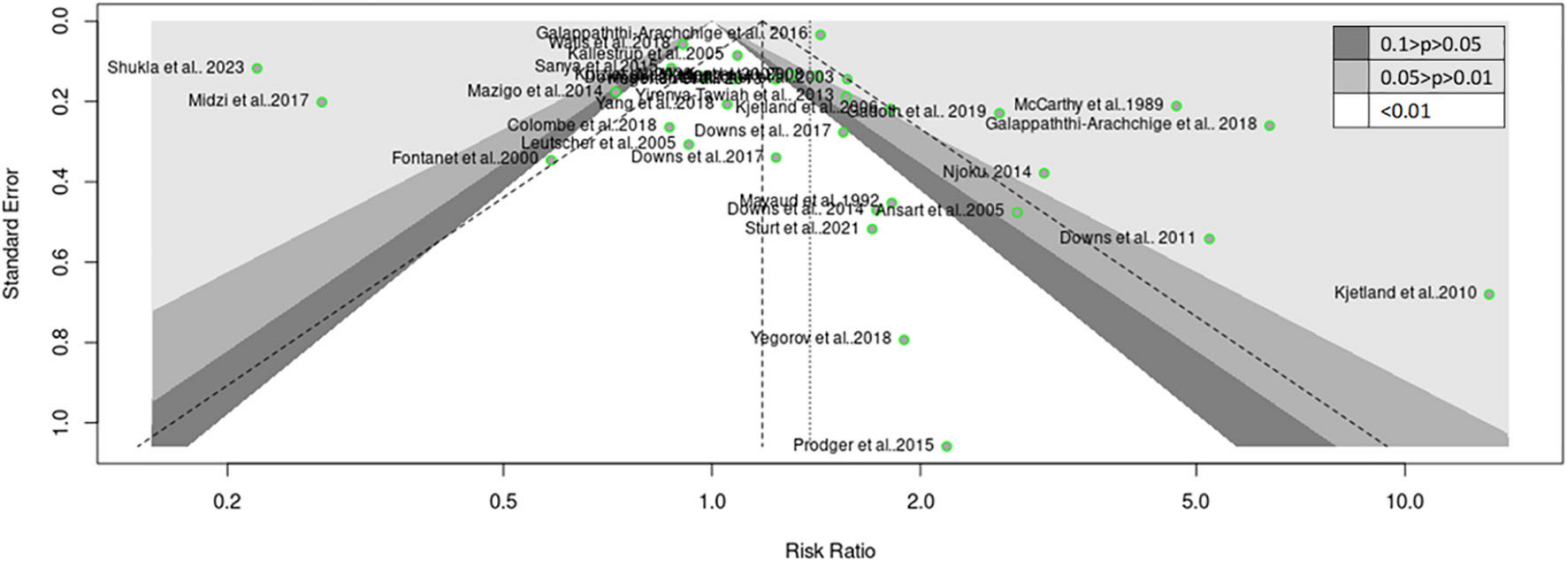
Funnel plot (Trim and Fill) showing asymmetrical distribution for human schistosomiasis and sexually transmitted infection from the 33 studies.

FGS has been closely linked with an increased risk of STIs, potentially mediated by schistosome-related tissue damage and immune responses. This association is particularly evident in regions endemic for schistosomiasis. The variability in schistosomiasis prevalence and its role in STIs could not be entirely explained by geographic differences alone, prompting an assessment of gender-specific influences on this heterogeneity. The subgroup analysis revealed that gender plays a significant role in the risk of STIs associated with schistosomiasis. The common effect model indicated a significant risk for females with a relative risk (RR) of 1.18 (95% CI: 1.13–1.23, z/t = 7.55, p<0.0001), while the random effect model also supported this with an RR of 1.38 (95% CI: 1.05–1.83, z/t = 2.39, p = 0.023). Significant heterogeneity among gender subgroups was noted (Q = 475, df = 32, p<0.0001). Detailed analysis showed that females had a notably higher risk of STIs in the context of schistosomiasis (k = 17, RR: 1.30, 95% CI: 1.23–1.37, Q = 316.78, I^2^ = 94.9%), compared to males (k = 6, RR: 0.94, 95% CI: 0.77–1.15, Q = 53.44, I^2^ = 90.6%) and the combined group of females and males (k = 9, RR: 0.95, 95% CI: 0.88–1.02, Q = 16.38, I^2^ = 50.2%) (**Figure 6**). The common effect model indicated heterogeneity both between groups (Q = 89.06, df = 3, p<0.0001) and within groups (Q = 386.59, df = 29, p<0.0001). Similarly, the random effect model for subgroup heterogeneity between groups also showed significant variation (Q = 53.33, df = 3, p<0.0001), with females having an RR of 1.72 (95% CI: 1.10–2.67), males having an RR of 1.00 (95% CI: 0.45–2.24), and the combined group having an RR of 0.96 (95% CI: 0.80–1.14) (**Figure 7**). Both the common and random effect models confirm that females have a higher risk of STIs in the context of schistosomiasis infection compared to males and the combined groups. This emphasizes the need for gender-specific approaches in managing schistosomiasis and related STIs, highlighting the critical role of addressing female genital health in endemic regions. These results suggest that individuals from West and Southern Africa had a higher risk of coinfections between schistosomiasis and STIs compared to inhabitants from East Africa. Similarly, females were the only with a higher risk of coinfections between schistosomiasis and STIs compared to males and the combined groups (males and females).In addition, these results were not affected by a publication bias. In detail, Fail-safe N calculation using the Rosenthal Approach showed significance in observed levels (p<0.0001) with the target levels (p = 0.05, Fail-safe N = 498) and funnel plot (Trim and Fill) displaying a graphical presentation of studies with no publication bias (**Figure 8**). Egger’s regression analysis for funnel plot asymmetry results confirmed the absence of a significant level of publication bias (t = 0.40, df = 31, p = 0.6947). The sample estimates showed bias (se. bias) = 0.3964 (1.005) with a multiplicative residual heterogeneity variance of (tau^2^ = 15.2663).

## Discussion

The coexistence of STIs such as chlamydia, gonorrhea, mycoplasma, and trichomoniasis infections among both men and women with *S. haematobium* infections highlights the interconnectedness of these diseases within populations living in endemic areas (McCarthy et al., 1989; Prodger et al., 2015; Nakagawa et al., 2022). The imbalance in focus on HIV coinfections due to factors like a disproportionate infection burden, perceived severity, and resources is due to a lack of a comprehensive disease assessment approach. Addressing coinfections with other STIs is crucial for improving health outcomes and addressing complex health needs in affected populations. This systematic review and meta-analysis aimed to elucidate the relationship between *Schistosoma* infections and STIs within populations living in areas endemic to schistosomiasis. By synthesizing existing research findings, the study sought to provide insights into the potential interactions between schistosomiasis and various STIs.

The results of this study showed that females are only gender that has a higher risk of STIs when one becomes infected with schistosoma species. Previous studies reported a significant association between HPV infection and FGS, suggesting a higher HPV infection likelihood, but *Chlamydia* was negatively associated with FGS, indicating a lower *Chlamydia* infection risk (Mbabazi et al., 2011; Prodger et al., 2015; Downs et al., 2017a; Colombe et al., 2018b). Another study reported a similarly high prevalence of FGS and *Chlamydia* infection, with both conditions affecting more than one-fifth of the young rural population studied (Kjetland et al., 2005; Dubbink et al., 2018). Another study conducted among expectant mothers showed that 17.4% of expectant mothers were infected with *Schistosoma haematobium*, of which 3.1% had *Chlamydia trachomatis*, 1.4% had *Neisseria gonorrhoeae*, and 14.6% had *Trichomonas vaginalis* (McCarthy et al., 1989). Interestingly, women infected with urogenital schistosomiasis were at significantly increased odds of harboring a *Chlamydia trachomatis*, *Neisseria gonorrhoeae*, or *Trichomonas vaginalis* infection, with an adjusted odds ratio of 3.0 and a 95% confidence interval of 1.5 to 6.0 (Chersich et al., 2018). However, it’s noteworthy that reports of clinical symptoms were low across these infections, ranging from 17.2% for schistosomiasis to 30.8% for *Trichomonas vaginalis* cases (McCarthy et al., 1989).

Indeed, there is evidence suggesting frequent coinfections between schistosomiasis and a range of other STIs such as syphilis, gonorrhea, chlamydia, trichomoniasis, HSV, HIV, and HPV (Downs et al., 2012; Korenromp et al., 2018; Migliavaca et al., 2020; Nakagawa et al., 2022). However, despite this evidence, attention has predominantly focused on the relationship between schistosomiasis and HIV, often neglecting the potential impact of coinfections with other STIs.

The pooled estimates from the 33 included published articles showed a significantly high risk of coinfections between schistosomiasis and STIs among people living in schistosomiasis-endemic communities. The previous study found that the overall proportions of individuals with HIV and syphilis in the population were 14.2% (95% CI: 12.2–16.4) and 15.6% (95% CI: 13.5–17.8), respectively. This population also exhibited a high prevalence of schistosome infections, with 85% of males and 80% of females being infected. Thus, there is significant overlap between schistosome infections and STIs such as HIV and syphilis (Gallagher et al., 2017; Zirimenya et al., 2020; Waheed et al., 2023). Similarly, there is a high overlap of infections such as gonorrhea, chlamydia, trichomoniasis, herpes HSV, HIV with schistosome infections (Leutscher et al., 2008; Downs et al., 2012; Downs et al., 2017a). This overlapping prevalence indicates that individuals in schistosomiasis-endemic areas are at significant risk of contracting multiple STIs simultaneously (Leutscher et al., 2008; Gallagher et al., 2017; Zirimenya et al., 2020; Leandro et al., 2021; Waheed et al., 2023).

The Sustainable Development Goals (SDGs) identified in the 2030 Agenda for Sustainable Development include Goal 3, which aims to end STI epidemics, which are major public health concerns (Leandro et al., 2021). Achieving this goal requires a comprehensive approach that will ensure universal health coverage and a continuum of services that encompass prevention, diagnosis, treatment, care for STIs, and making these services accessible to everyone without financial hardship. The key targets include a 90% reduction in the global incidence of *Treponema pallidum* and *Neisseria gonorrhoeae* from 2018 levels and reducing congenital syphilis to ≤ 50 cases per 100,000 live births in 80% of countries (Aula et al., 2021). Additionally, sustaining high coverage of the HPV vaccine at the national and district levels is crucial. The ultimate aim is to achieve zero new infections, complications, deaths, and discrimination related to STIs with free and easy access to prevention and treatment services, thereby enabling people to live long and healthy lives (Kayuni et al., 2019; Ogongo et al., 2022). This necessitates strong health systems, political commitment, adequate funding, cross-sector collaboration, and addressing social determinants of health, education, and stigma reduction.

Laboratory confirmation of schistosomiasis and STIs has provided objective evidence of the increased risk of STIs in individuals with schistosomiasis (McCarthy et al., 1989; Ndhlovu et al., 2007; Downs et al., 2012; Kayuni et al., 2019; Honkpehedji et al., 2020). This highlights that relying solely on syndromic management and focusing only on HIV-schistosome coinfection may not be sufficient. Diagnosing FGS presents significant challenges due to the anatomical and pathological characteristics of the disease (Vaillant et al., 2024). Schistosome eggs tend to become lodged in the vaginal and cervical tissues, making their detection in urine samples difficult and often unreliable (Kim et al., 2023). Traditional diagnostic methods, such as the Kato-Katz technique and egg microscopy, are insufficient for FGS, particularly in cases with low egg burden, where egg detection sensitivity is substantially reduced (World Health Organization, 2008; Jarolimova et al., 2022). Recent advancements in diagnostic technologies, particularly the detection of circulating cathodic antigen (CCA) and circulating anodic antigen (CAA), offer new possibilities for improving FGS diagnosis (Kenyon et al., 2014; Zheng et al., 2022). The CCA test has shown promise in detecting Schistosoma mansoni infections with relatively high sensitivity and specificity (Kenyon et al., 2016). However, this test has proven less effective for diagnosing Schistosoma haematobium, the primary cause of FGS, due to lower sensitivity in detecting this species (Kenyon et al., 2016). Given these limitations, there is a pressing need for the development of novel diagnostic tools or the enhancement of existing antigen-based tests to improve their sensitivity and specificity for S. haematobium and other schistosome species. Such improvements are crucial for accurately diagnosing FGS and facilitating the effective control and elimination of neglected tropical diseases (NTDs) in line with the SDGs. Enhanced diagnostic methods will play a pivotal role in identifying and treating affected individuals, thereby reducing disease transmission and advancing public health objectives globally. Comprehensive diagnostic approaches are necessary to identify and manage the full spectrum of STIs in schistosomiasis-endemic populations, thereby improving overall health outcomes and addressing the broader public health challenges posed by these coexisting infections.

Subgrouping the articles by geographical regions revealed that study participants in schistosomiasis-endemic regions of West Africa and Southern Africa had a significantly higher risk of coinfections between schistosomiasis and STIs. Conversely, in East Africa, there were lower risks of such coinfections. Although schistosomiasis has been reported as a significant risk factor for HIV in East Africa, especially in Tanzania, the prevalence of chlamydia and other STIs (excluding HIV) is relatively low, estimated at around 3% among individuals aged 15–49 years in clinical or community settings, and 10% in high-risk groups in East Africa (Seidu et al., 2020). Notably, chlamydia prevalence is lower in East Africa (2.8%) compared to Southern (12.5%) and West/Central Africa (19.1%) (Bassey et al., 2022). This geographic variation in STI prevalence could explain why this study observed a lower risk of schistosomiasis-associated STIs in East Africa compared to West and Southern Africa. Several factors may contribute to this phenomenon, including the lower endemicity of STI pathogens (other than HIV) in East Africa, distinct behavioral characteristics of individuals and communities, and the healthcare services provided in the region. These factors combined may influence the overall lower risk of STIs in the context of schistosomiasis in East Africa. This regional variation highlights the importance of considering local epidemiological factors and the unique context of each region when designing interventions and public health strategies to address the coexistence of schistosomiasis and STIs (Aula et al., 2021). Tailoring interventions to the specific needs and challenges of each region can lead to more effective prevention and management of these coinfections, ultimately improving health outcomes in schistosomiasis-endemic areas.

The previous study identified significant variation in STI incidence and prevalence across different global regions, with a positive correlation coefficient (Kenyon et al., 2014). East Asia/Pacific and North Africa/Middle East were categorized in the lowest tier, while Sub-Saharan Africa was placed in the highest tier (Kenyon et al., 2014; Lealem et al., 2024). This categorization reflects the diverse epidemiological landscape of STIs worldwide, with some regions experiencing higher burdens of STIs than others. Studies from various countries, including Ethiopia, Kenya, South Africa, Uganda, the UK, and the US, have consistently shown that ethnic groups with high HIV prevalences also tend to have higher prevalences of other STIs (Kenyon et al., 2016; Kenyon et al., 2017). For example, in the US, non-Hispanic black populations have been found to have higher prevalences of HSV-2, chlamydia, gonorrhea, syphilis, and trichomoniasis (National Academies of Sciences, Engineering, and Medicine et al., 2021). These findings underscore the importance of considering socio-demographic factors, such as ethnicity, in understanding and addressing health inequities related to STIs. Effective interventions should be tailored to address the specific needs of diverse populations and target the underlying social determinants of health contributing to these disparities. These reports support the observation that the risks of the coexistence of schistosomiasis and STIs vary from place to place.

## Conclusion

**Figure 9:**
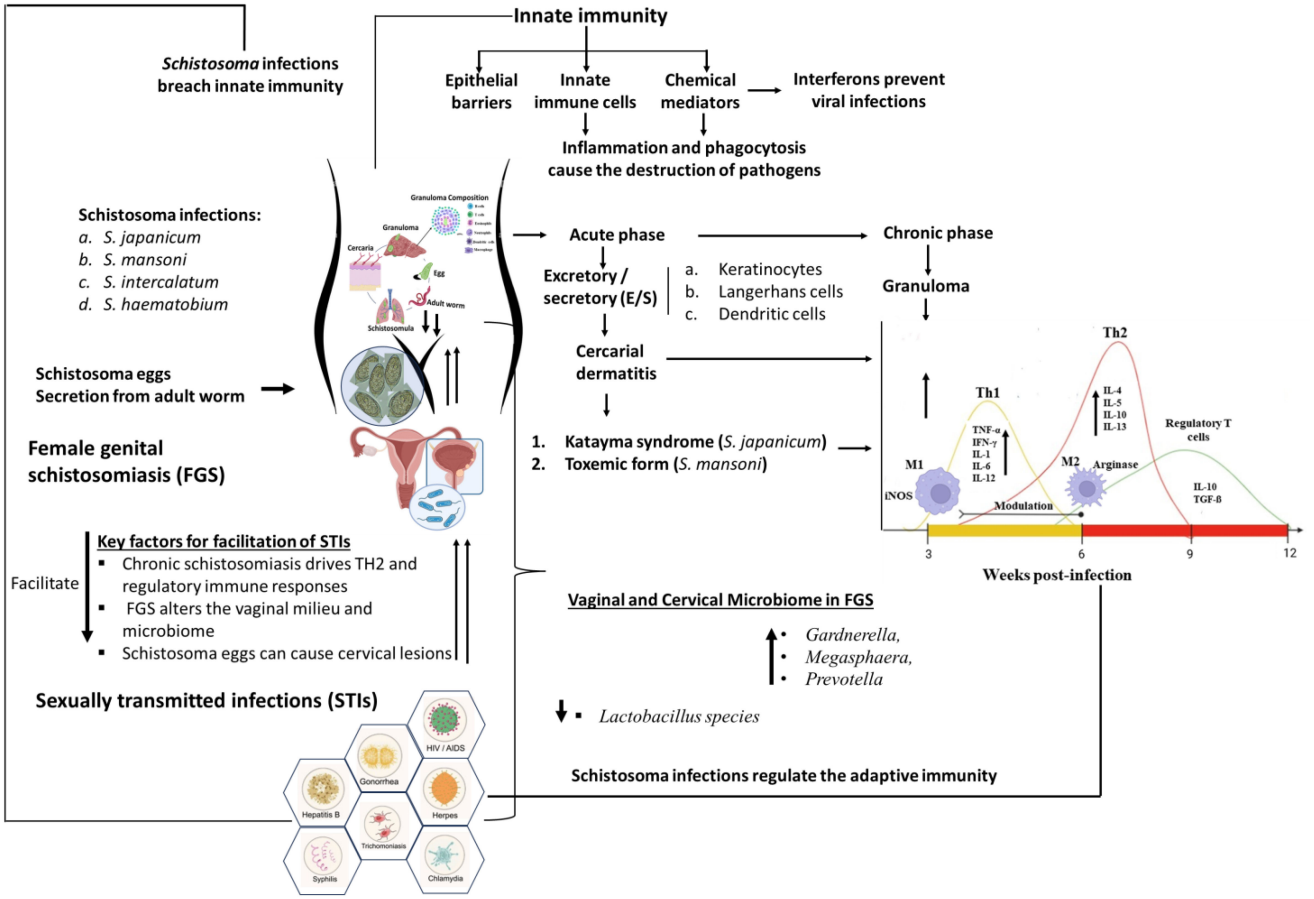
Diagram showing the mechanisms involved in *Schistosoma* infection modulate the immune system to enhance coinfection with STIs. Schistosoma infections, including *S. japonicum*, *S. mansoni, S. intercalatum, and S. haematobium*, significantly modulate the immune system, enhancing coinfection with STIs. These parasites breach innate immunity by releasing excretory/secretory (E/S) molecules that activate keratinocytes, Langerhans cells, and dendritic cells, leading to conditions such as cercarial dermatitis. Specific manifestations include Katayama syndrome in *S. japonicum* infections and toxemic forms in *S. mansoni* infections. This disruption of the innate immune system progresses to a modulation of the immune response from a TH1 response in the acute phase to a TH2 response and increased regulatory T cells in the chronic phase. Immunity against STIs requires a functional TH1 immune response, which plays a crucial role in the body's defense mechanism by activating macrophages and stimulating the production of certain cytokines that promote the elimination of intracellular pathogens. A robust TH1 response helps control and clear infections by producing IFN-γ and other pro-inflammatory cytokines, which enhance the ability of immune cells to target and destroy infected cells. In the context of schistosomiasis, however, the immune response often shifts from a TH1 to a TH2 and regulatory T cell-dominated response during chronic infection, which can compromise the body's ability to effectively combat co-infections with STIs. This shift can hinder the immune system's capacity to mount an adequate TH1 response, thereby facilitating the persistence and transmission of STIs in affected individuals. Such immune modulation mechanisms might facilitate the transmission and colonization of STIs in individuals with schistosomiasis. The secretion of eggs by Schistosoma parasites can cause lesions in the genital tract, which may facilitate the transmission and acquisition of STIs, particularly in FGS. FGS causes changes in the vaginal milieu and microbiome, with schistosome egg secretion from adult worms causing cervical lesions that further enhance STI acquisition. These lesions compromise the mucosal barrier, allowing easier entry and establishment of pathogens. Additionally, changes in the vaginal flora and microbiome, such as a decrease in protective lactobacilli, can increase susceptibility to infections like bacterial vaginosis, further enhancing the risk of acquiring STIs. The coinfection of Schistosoma and STIs results in complications that increase disease severity and pose significant challenges in treatment, control, and management, highlighting the intricate interplay between parasitic infections and STIs in exacerbating health outcomes.

**Figure 10:**
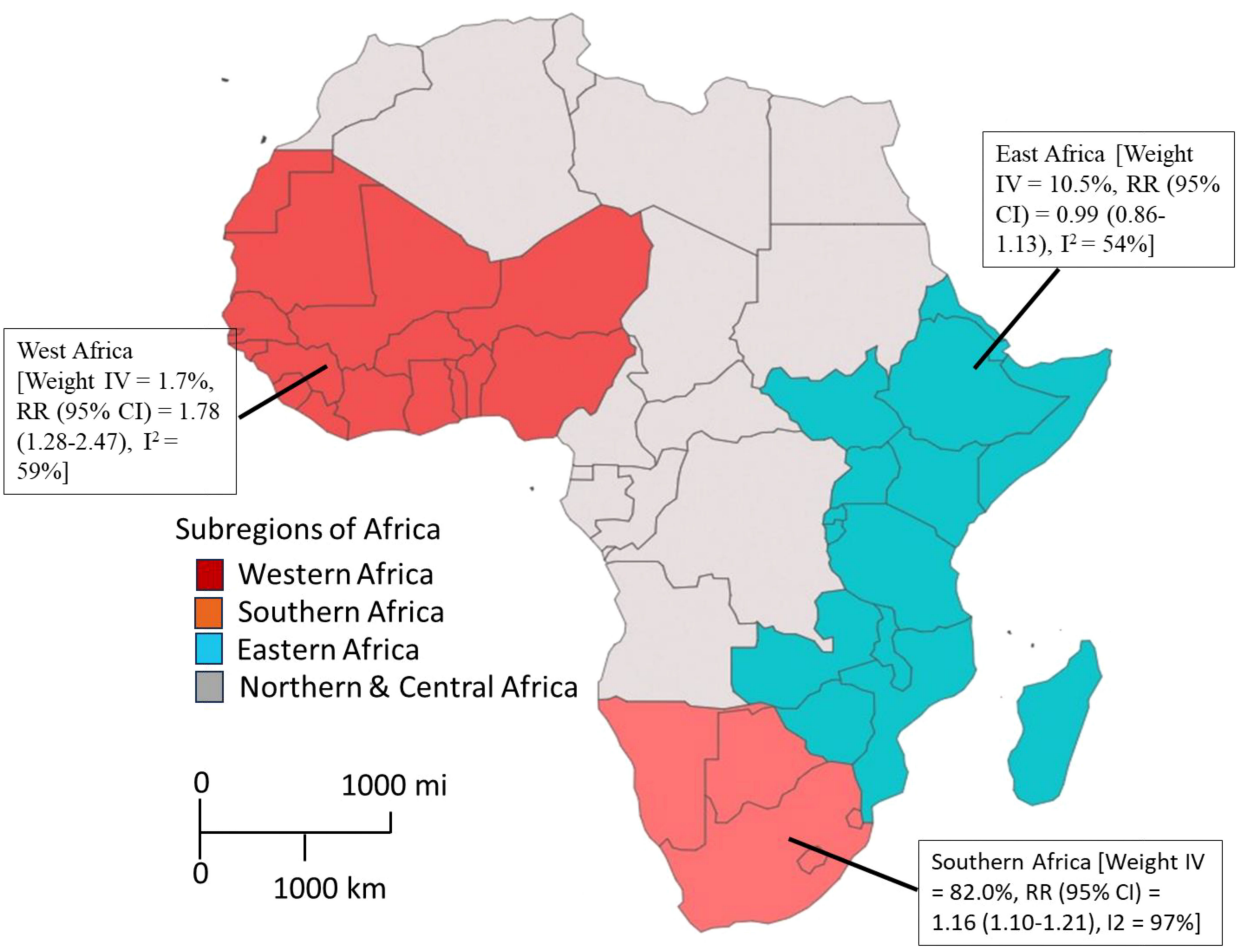
The Risk of coinfection of schistosomiasis and STIs across three geographical areas in Africa. Individuals from West and Southern Africa had a higher risk of coinfections between schistosomiasis and STIs compared to inhabitants from East Africa.

The pooled estimates from the 33 included published articles showed a significantly high risk of coinfections between schistosomiasis and STIs among people living in schistosomiasis-endemic communities. **Figure 9** highlights the role Schistosoma plays in facilitating infection and colonization of STIs. The study found that participants in West and Southern Africa had a higher risk of coinfections, while those in East Africa had lower risks (**Figure 10**). Comprehensive diagnostic approaches are crucial for identifying and managing STIs in schistosomiasis-endemic populations. This will improve health outcomes, address public health challenges, reduce disease burden, and help achieve universal health coverage as stated in SDG 3.

## Data Availability

The original contributions presented in the study are included in the article/**Supplementary Material**. Further inquiries can be directed to the corresponding authors.
