# Supplementary material for: Schistosomiasis endemicity and its role in sexually transmitted infections – a systematic review and meta-analysis
Source: Front Parasitol. 2024 Sep 2;3:1451149. doi: 10.3389/fpara.2024.1451149 (PMC11731636; doi:10.3389/fpara.2024.1451149)
Supplement: Supplementary file 1 [file Table1.doc]

S1 Table: PRISMA Checklist

| **Section/topic** | **#** | **Checklist item** | **Reported on page #** |
| --- | --- | --- | --- |
| **TITLE** | | |  |
| Title | 1 | Identify the report as a systematic review, meta-analysis, or both. |  |
| **ABSTRACT** | | |  |
| Structured summary | 2 | Provide a structured summary including, as applicable: background; objectives; data sources; study eligibility criteria, participants, and interventions; study appraisal and synthesis methods; results; limitations; conclusions and implications of key findings; systematic review registration number. |  |
| **INTRODUCTION** | | |  |
| Rationale | 3 | Describe the rationale for the review in the context of what is already known. |  |
| Objectives | 4 | Provide an explicit statement of questions being addressed with reference to participants, interventions, comparisons, outcomes, and study design (PICOS). |  |
| **METHODS** | | |  |
| Protocol and registration | 5 | Indicate if a review protocol exists, if and where it can be accessed (e.g., Web address), and, if available, provide registration information including registration number. |  |
| Eligibility criteria | 6 | Specify study characteristics (e.g., PICOS, length of follow-up) and report characteristics (e.g., years considered, language, publication status) used as criteria for eligibility, giving rationale. |  |
| Information sources | 7 | Describe all information sources (e.g., databases with dates of coverage, contact with study authors to identify additional studies) in the search and date last searched. |  |
| Search | 8 | Present full electronic search strategy for at least one database, including any limits used, such that it could be repeated. |  |
| Study selection | 9 | State the process for selecting studies (i.e., screening, eligibility, included in systematic review, and, if applicable, included in the meta-analysis). |  |
| Data collection process | 10 | Describe method of data extraction from reports (e.g., piloted forms, independently, in duplicate) and any processes for obtaining and confirming data from investigators. |  |
| Data items | 11 | List and define all variables for which data were sought (e.g., PICOS, funding sources) and any assumptions and simplifications made. |  |
| Risk of bias in individual studies | 12 | Describe methods used for assessing risk of bias of individual studies (including specification of whether this was done at the study or outcome level), and how this information is to be used in any data synthesis. |  |
| Summary measures | 13 | State the principal summary measures (e.g., risk ratio, difference in means). |  |
| Synthesis of results | 14 | Describe the methods of handling data and combining results of studies, if done, including measures of consistency (e.g., I^2^) for each meta-analysis. |  |

Page 1 of 2

| **Section/topic** | **#** | **Checklist item** | **Reported on page #** |
| --- | --- | --- | --- |
| Risk of bias across studies | 15 | Specify any assessment of risk of bias that may affect the cumulative evidence (e.g., publication bias, selective reporting within studies). |  |
| Additional analyses | 16 | Describe methods of additional analyses (e.g., sensitivity or subgroup analyses, meta-regression), if done, indicating which were pre-specified. |  |
| **RESULTS** | | |  |
| Study selection | 17 | Give numbers of studies screened, assessed for eligibility, and included in the review, with reasons for exclusions at each stage, ideally with a flow diagram. |  |
| Study characteristics | 18 | For each study, present characteristics for which data were extracted (e.g., study size, PICOS, follow-up period) and provide the citations. |  |
| Risk of bias within studies | 19 | Present data on risk of bias of each study and, if available, any outcome level assessment (see item 12). |  |
| Results of individual studies | 20 | For all outcomes considered (benefits or harms), present, for each study: (a) simple summary data for each intervention group (b) effect estimates and confidence intervals, ideally with a forest plot. |  |
| Synthesis of results | 21 | Present results of each meta-analysis done, including confidence intervals and measures of consistency. |  |
| Risk of bias across studies | 22 | Present results of any assessment of risk of bias across studies (see Item 15). |  |
| Additional analysis | 23 | Give results of additional analyses, if done (e.g., sensitivity or subgroup analyses, meta-regression [see Item 16]). |  |
| **DISCUSSION** | | |  |
| Summary of evidence | 24 | Summarize the main findings including the strength of evidence for each main outcome; consider their relevance to key groups (e.g., healthcare providers, users, and policy makers). |  |
| Limitations | 25 | Discuss limitations at study and outcome level (e.g., risk of bias), and at review-level (e.g., incomplete retrieval of identified research, reporting bias). |  |
| Conclusions | 26 | Provide a general interpretation of the results in the context of other evidence, and implications for future research. |  |
| **FUNDING** | | |  |
| Funding | 27 | Describe sources of funding for the systematic review and other support (e.g., supply of data); role of funders for the systematic review. |  |

S2 Table: Literature Search Strategy

| **No** | **Database** | **Search query** | **Hits** | **Filters** |
| --- | --- | --- | --- | --- |
| 1 | PubMed | (("schistosomiasis"[MeSH Terms] OR "schistosomiasis"[All Fields] OR "schistosomiases"[All Fields]) AND ("infect"[All Fields] OR "infectability"[All Fields] OR "infectable"[All Fields] OR "infectant"[All Fields] OR "infectants"[All Fields] OR "infected"[All Fields] OR "infecteds"[All Fields] OR "infectibility"[All Fields] OR "infectible"[All Fields] OR "infecting"[All Fields] OR "infection s"[All Fields] OR "infections"[MeSH Terms] OR "infections"[All Fields] OR "infection"[All Fields] OR "infective"[All Fields] OR "infectiveness"[All Fields] OR "infectives"[All Fields] OR "infectivities"[All Fields] OR "infects"[All Fields] OR "pathogenicity"[MeSH Subheading] OR "pathogenicity"[All Fields] OR "infectivity"[All Fields]) AND ("sexually transmitted diseases"[MeSH Terms] OR ("sexually"[All Fields] AND "transmitted"[All Fields] AND "diseases"[All Fields]) OR "sexually transmitted diseases"[All Fields] OR ("sexually"[All Fields] AND "transmitted"[All Fields] AND "infections"[All Fields]) OR "sexually transmitted infections"[All Fields])) AND ((ffrft[Filter]) AND (fha[Filter]) AND (fft[Filter])) Translations schistosomiasis: "schistosomiasis"[MeSH Terms] OR "schistosomiasis"[All Fields] OR "schistosomiases"[All Fields] infection: "infect"[All Fields] OR "infectability"[All Fields] OR "infectable"[All Fields] OR "infectant"[All Fields] OR "infectants"[All Fields] OR "infected"[All Fields] OR "infecteds"[All Fields] OR "infectibility"[All Fields] OR "infectible"[All Fields] OR "infecting"[All Fields] OR "infection's"[All Fields] OR "infections"[MeSH Terms] OR "infections"[All Fields] OR "infection"[All Fields] OR "infective"[All Fields] OR "infectiveness"[All Fields] OR "infectives"[All Fields] OR "infectivities"[All Fields] OR "infects"[All Fields] OR "pathogenicity"[Subheading] OR "pathogenicity"[All Fields] OR "infectivity"[All Fields] sexually transmitted infections: "sexually transmitted diseases"[MeSH Terms] OR ("sexually"[All Fields] AND "transmitted"[All Fields] AND "diseases"[All Fields]) OR "sexually transmitted diseases"[All Fields] OR ("sexually"[All Fields] AND "transmitted"[All Fields] AND "infections"[All Fields]) OR "sexually transmitted infections"[All Fields] | 172 | Abstract, Free full text, Full text, (1975 to 2024) |
| 2 | Scopus | TITLE-ABS-KEY ( schistosomiasis AND infection AND sexually AND transmitted AND infections ) AND PUBYEAR > 1974 AND PUBYEAR < 2025 AND ( LIMIT-TO ( DOCTYPE, "ar" ) ) | 104 | Limited articles, (search from 1975 to 2024) |
| 3 | MedlinePlus | Schistosomiasis infection and sexually transmitted infections | 4 |  |
| 4 | ScienceDirect | Schistosomiasis infection and sexually transmitted infections | 234 | Search from 1975 to 2024 |
| 5 | Cochrane | Trials matching schistosomiasis infection and sexually transmitted infections in Title Abstract Keyword - (Word variations have been searched) Did you mean: schistosomiases schistosomiasic schistosomosis Cochrane Central Register of Controlled Trials | 2 |  |
| 6 | ClinicalTrials.gov | Schistosomiasis infection and sexually transmitted infections | 4 |  |
|  | Total |  | 520 |  |

**S3 Table:** **Quality assessment results**

|  |  |  | **Quality criteria score** | | | | | | | | |  |
| --- | --- | --- | --- | --- | --- | --- | --- | --- | --- | --- | --- | --- |
| **SN** | **Study** | **country** | **A** | **B** | **C** | **D** | **E** | **F** | **G** | **H** | **I** | **Total** |
| 1 | Mayaud et al.,1992 [38] | Tanzania | 1 | 1 | 1 | 1 | 1 | 1 | 1 | 1 | 1 | 9 |
| 2 | Ansart et al.,2005 [39] | France | 1 | 1 | 1 | 1 | 1 | 1 | 1 | 1 | 1 | 9 |
| 3 | Kjetland et al.,2008 [40] | Zimbabwe | 1 | 1 | 1 | 1 | 1 | 1 | 1 | 1 | 1 | 9 |
| 4 | Hegertun et al., 2013 [41] | South Africa | 1 | 1 | 1 | 1 | 1 | 0 | 1 | 1 | 1 | 8 |
| 5 | Downs et al., 2014 [42] | Tanzanian | 1 | 1 | 1 | 1 | 1 | 1 | 1 | 1 | 1 | 9 |
| 6 | Galappaththi-Arachchige et al., 2016 [43] | South Africa | 1 | 1 | 1 | 1 | 1 | 1 | 1 | 1 | 1 | 9 |
| 7 | Galappaththi-Arachchige et al., 2018 [44] | South Africa | 1 | 1 | 1 | 1 | 1 | 1 | 1 | 1 | 1 | 9 |
| 8 | Yegorov et al.,2018 [45] | Uganda | 1 | 1 | 1 | 1 | 1 | 1 | 1 | 1 | 1 | 9 |
| 9 | Gadoth et al., 2019 [46] | Democratic Republic of Congo | 1 | 1 | 1 | 1 | 1 | 1 | 1 | 1 | 1 | 9 |
| 10 | Kjetland et al.,2006 [47] | Zimbabwe | 1 | 1 | 1 | 1 | 1 | 1 | 1 | 1 | 1 | 9 |
| 11 | Downs et al., 2011 [48] | Tanzania | 1 | 1 | 1 | 1 | 1 | 1 | 1 | 1 | 1 | 9 |
| 12 | Downs et al., 2017 [49] | Tanzania | 1 | 1 | 1 | 1 | 1 | 1 | 1 | 1 | 1 | 9 |
| 13 | Shukla et al., 2023 [50] | South Africa | 1 | 1 | 1 | 0 | 1 | 1 | 1 | 1 | 1 | 8 |
| 14 | Leutscher et al.,2003 [51] | Madagascar | 1 | 1 | 1 | 1 | 1 | 1 | 1 | 1 | 1 | 9 |
| 15 | McCarthy et al.,1989 [52] | Sudan | 1 | 1 | 1 | 0 | 1 | 1 | 1 | 0 | 1 | 7 |
| 16 | [Downs et al., 2012](https://pubmed.ncbi.nlm.nih.gov/?term=Downs JA%5BAuthor%5D) [53] | Tanzania | 1 | 1 | 1 | 1 | 1 | 1 | 1 | 1 | 1 | 9 |
| 17 | Walls et al.,2018 [54] | Zambia | 1 | 1 | 1 | 1 | 1 | 1 | 1 | 1 | 1 | 9 |
| 18 | Colombe et al.,2018 [55] | Tanzania | 1 | 1 | 1 | 1 | 1 | 1 | 1 | 1 | 1 | 9 |
| 19 | Downs et al.,2017 [56] | Tanzania | 1 | 1 | 1 | 1 | 1 | 1 | 1 | 1 | 1 | 9 |
| 20 | Sanya et al.2015 [57] | Ugandan | 1 | 1 | 1 | 1 | 1 | 1 | 1 | 1 | 1 | 9 |
| 21 | Fontanet et al.,2000 [58] | Ethiopia | 1 | 1 | 1 | 1 | 1 | 1 | 1 | 1 | 1 | 9 |
| 22 | Ndhlovu et al.,2007 [59] | Zimbabwe | 1 | 1 | 1 | 1 | 1 | 1 | 1 | 1 | 1 | 9 |
| 23 | Midzi et al.,2017 [60] | Zimbabwe | 1 | 1 | 1 | 1 | 1 | 1 | 1 | 1 | 1 | 9 |
| 24 | Mazigo et al.,2014 [61] | Tanzania | 1 | 1 | 1 | 1 | 1 | 1 | 1 | 1 | 1 | 9 |
| 25 | Kjetland et al.,2010 [62] | Zimbabwe | 1 | 1 | 1 | 1 | 1 | 1 | 1 | 1 | 1 | 9 |
| 26 | Leutscher et al.,2005 [63] | Madagascar | 1 | 1 | 1 | 1 | 1 | 1 | 1 | 1 | 1 | 9 |
| 27 | Sturt et al.,2021 [64] | Zambia | 1 | 1 | 1 | 1 | 1 | 1 | 1 | 1 | 1 | 9 |
| 28 | Yirenya-Tawiah et al., 2013 [65] | Ghana | 1 | 1 | 1 | 1 | 1 | 1 | 1 | 1 | 1 | 9 |
| 29 | Kallestrup et al.,2005 [66] | Zimbabwe | 1 | 1 | 1 | 1 | 1 | 1 | 1 | 1 | 1 | 9 |
| 30 | Kutz et al., 2023 [67] | Madagascar | 1 | 1 | 1 | 1 | 1 | 1 | 1 | 1 | 1 | 9 |
| 31 | Yang et al.,2018 [68] | southwestern China | 1 | 1 | 1 | 0 | 1 | 1 | 1 | 1 | 1 | 8 |
| 32 | Njoku, 2014 [69] | Nigeria | 1 | 1 | 1 | 1 | 1 | 1 | 1 | 1 | 1 | 9 |
| 33 | Prodger et al.,2015 [70] | Ugandan | 1 | 1 | 1 | 1 | 1 | 1 | 1 | 1 | 1 | 9 |

**Definition of terms**: **A**; appropriateness of sample frame to address the target population, **B**; appropriateness of the way used to sample study participants, **C**; adequateness of sample size, **D**; description of study subjects and settings, **E**; data analysis coverage in the identified sample, **F**; validity of method used to identify schistosomiasis reinfection rate, **G**; reliability of method used to measure schistosomiasis reinfection rate for all participants, **H**; appropriateness of statistical tests used in data analysis and **I**; adequateness of response rate

S4. Table: Joanna Briggs Institute critical appraisal checklist_guidelines for Quality assessment

| **Term** |  | **Definition** |
| --- | --- | --- |
| Critical appraisal |  | The process of systematically assessing the outcome of scientific research to judge its trustworthiness, value, and relevance in a particular context. |
| External validity |  | Applicability of the findings to a given population. |
| Generalizability |  | The degree to which the results of a study can be applied to a broader population or situation. |
| Imprecision |  | The GRADE approach to rating imprecision focuses on the 95% CI around the best estimate of the absolute effect. |
| Indirectness |  | The GRADE approach to rating indirectness focuses on concerns about how the population, intervention, or outcomes differ from those of interest. |
| Internal validity |  | A measure of how well a study is conducted and how accurately its results reflect the studied group. |
| Methodological quality |  | The extent to which there is potential for errors and bias in the design and execution of a study. |
| Power |  | The probability of finding a statistically significant result. |
| Publication bias |  | The likelihood studies have not been published based on the outcome of the research study. |
| Random error |  | An error in measurement caused by factors that vary from one measurement to another. |
| Reporting quality |  | The extent to which a complete and transparent description of the design, conduct, and analysis of a study is given. |
| Risk of bias |  | The likelihood that features of the study design or conduct of the study will give misleading results. |
| Sample size |  | The number of participants or observations included in a study. |
| Statistical conclusion validity |  | The extent to which the conclusions of research are founded on adequate analysis of the data. |
| Systematic error |  | Errors that affect the accuracy of a measurement or cause readings to differ from the true value by a consistent amount each time a measurement is made. |

S5. Table: A detail characteristics of eligible studies with study methods and prevalence of reported STDs

| **Study** | country | STD | N | Methods |
| --- | --- | --- | --- | --- |
| Mayaud et al.,1992 [38] | Tanzania | Neisseria gonorrhoeae/Chlamydia trachomatis [S. haematobium-STD, 2/55 (3.6%); S. haematobium, 10/151 (7%); S. haematobium, 6/42 (14%); N. gonorrhoeae, 2/151 (1.3%), C. trachomatis, 2/151 (1.3%), NSU, 12/151 (8%)] | 248 | In July 1991, a study was conducted at Sekou Toure Hospital in Mwanza Region, Tanzania. Male patients aged 15-54 were eligible. Urine specimens were tested for schistosomiasis using a LE dipstick and ELISA. The study focused on diagnosing schistosomiasis using qualitative methods. |
| Ansart et al.,2005 [39] | France | [STDs, 22/622 (3.4%); Schistosomiasis, 46/622 (7.2%) ] | 622 | This study included adult patients in Paris, France, with travel-related diseases, seroconversion for schistosomiasis, or schistosoma egg appearances during 2002-2003. |
| Kjetland et al.,2008 [40] | Zimbabwe | [HIV (29% positive, 153 ⁄ 523; herpes simplex virus type 2 (HSV-2; 65%, 307 ⁄ 476); Current syphilis (3%, 13/479); Past syphilis, 6% (30 ⁄ 481) human papillomavirus (HPV; 33%, 81 ⁄ 236; Neisseriae gonorrhoeae (0.9%, 4⁄429); Chlamydia trachomatis (1.4%, 6 ⁄ 429); S. haematobium ova in genital tissue: in Pap smears (6%, 26 ⁄ 437), wet mounts (40%, 37 ⁄ 92), biopsies (61%, 17 ⁄ 28), ﬁltration and examined by microscope for ova; 40% (178 ⁄ 451] | 483 | The study involved women aged 20-49 in North Western Zimbabwe, who underwent vaginal lavage, photocolposcopic examination, and Papanicolaou (Pap) smears. The presence of S. haematobium ova in genital tissue was confirmed using Pap smears, wet mounts, and biopsies. Serologic tests were conducted for HIV, HSV-2, and trichomonas. Current syphilis was diagnosed by seroconversion, while past syphilis was found in 6%. Trichomoniasis, candidiasis, Neisseriae gonorrhoeae, bacterial vaginosis, and Chlamydia trachomatis were tested. Chi-squared and Fisher's exact tests were used to study the association between clinical pathology in the genitals and patient complaint. |
| Hegertun et al., 2013 [41] | South Africa | [HIV 3/980 (0.3%);  S. haematobium eggs 32% (312/ 970)] | 1057 | A cross-sectional study was conducted in 18 primary schools in rural Ugu District, South Africa, involving girls aged 10-12. Urine samples were collected and analyzed for S. haematobium, a endemic fungus, and analyzed for Ascaris lumbricoides, Ancylostoma duodenale, Taenia solium, Trichuris trichiura, and S. mansoni. |
| Downs et al., 2014 [42] | Tanzanian | [Chlamydia, 4/33 (12%), Syphilis, 1/33 (3%), Gonorrhea, 1/33 (3%), Trichomoniasis, 1/33 (3%), Schistosomiasis, 39/39 (100%)] | 39 | A six-month cohort study involved women with Schistosoma haematobium infection in Lubiri and Nyamilama villages. They were treated with praziquantel and retested after six months. The study involved urine samples collected, filtered, and examined for schistosome ova, with serum tested for syphilis using Rapid Plasma Reagin and Treponema pallidum particle agglutination. |
| Galappaththi-Arachchige et al., 2016 [43] | South Africa | [C.trachomatis, 26.7% (217/814), T. vaginalis (19.8%, 169/853), N. gonorrhoea (12.0%, 98/814). Syphilis prevalence of 2.1% (18/858), C. albicans was detected in 13.5% (111/823), Bacterial vaginosis in 62.1% (484/779); S. haematobium ova 30.5% (242/794)] | 883 | A study was conducted in schools in KwaZulu-Natal, South Africa, from 2011 to 2013. Female students aged 16 and above were informed about schistosomiasis, and consenting young women aged 16-22 were invited for gynaecological examinations. STI analyses included cervico-vaginal lavage (CVL) and Papanicolaou (Pap) smears. Neisseria gonorrhoea, Chlamydia trachomatis, Trichomonas vaginalis, and bacterial vaginosis were detected using various tests. Herpes simplex type 2 antibodies were detected in serum using ELISA. |
| Galappaththi-Arachchige et al., 2018 [44] | South Africa | [HIV prevalence, 17.1% (241/1351); Chlamydia trachomatis, 24.8% (328/1325); Trichomonas vaginalis, 17.9% (245/1371); Neisseria gonorrhoea 10.9% (145/1325); Treponema pallidum, 1.8% (24/1350); schistosomiasis, 35.2% (496/1410) ] | 1413 | A cross-sectional study on female genital schistosomiasis in rural KZN, South Africa, involved high-school students. The study involved a structured questionnaire, visual inspection of the vulva, vagina, and cervix, and photocolposcopic examination. A positive diagnosis was given if the lesions were characteristic. The CVL was analyzed for Neisseria gonorrhoea and Chlamydia trachomatis, HIV testing was performed, and syphilis screening was performed. |
| Yegorov et al.,2018 [45] | Uganda | [HSV-2, 58.6% (34/58); STI, 12.1% (7/58);  T.vaginalis, 1.7% (1/58); Trachomatis, 8.6% (5/58); Gonorrhoeae, 1.7% (1/58); HSV-2-schistosomiasis, 63.6% (21/33); HSV-2-schistosomiasis, 63.6% (21/33); STI-schistosomiasis, 15.2% (5/33); Trachomatis - schistosomiasis, 15.2% (5/33).] | 58 | The study in Entebbe, Lake Victoria, diagnosed schistosomiasis in 58 consenting women aged 18-45. Urine samples were tested for Chlamydia trachomatis, Neisseria gonorrhoeae, Trichomonas vaginalis, and bacterial vaginosis using Roche Cobas PCR, OSOM rapid test, and Nugent criteria. |
| Gadoth et al., 2019 [46] | Democratic Republic of Congo | [Schistosomiasis coinfection with: Any STI, 21/63 (33.3%), Trichomonas vaginalis, 15/63 (23.8%), Chlamydia trachomatis, 2/62 (3.2%), Neisseria gonorrhoeae, 4/62 (6.5%), Chlamydia trachomatis/Neisseria gonorrhoeae, 6/ 62 (9.7%); Infections without schistosomiasis: Any STI, 44/296 (14.9%), Trichomonas vaginalis, 37/294 (12.6%), Chlamydia trachomatis, 9/289 (3.1%), Neisseria gonorrhoeae, 1/289 (0.3%), Chlamydia trachomatis/Neisseria gonorrhoeae, 10/289 (3.5%)] | 367 | A prospective cohort study was conducted on pregnant women in Kisantu health zone, Kongo Central Province, DRC, between 2016 and 2017. Participants were 18 years or older, between 4 and 35 weeks pregnant, and tested for Schistosoma haematobium (S. haematobium) using urine samples and vaginal swab specimens. Results were analyzed for CT, NG, and TV using DNA assay. Women positive for CT, NG, or TV were provided treatment and followed up for cure tests. |
| Kjetland et al.,2006 [47] | Zimbabwe | [HIV-Schistosomiasis coinfection,41% (29/70); HIV positive in the schistosomal ova negative group 26% (96/375)] | 445 | A cross-sectional study in Zimbabwe involved women aged 20-49, who were examined for sexually transmitted diseases (STDs) and cancer. Pap smears, wet mounts, and biopsies were used to diagnose genital issues. Serologic tests were conducted for HIV, trichomonas, and herpes simplex virus type 2. HSV-2 was found in 65% of cases. Other tests included cultures for Haemophilus ducreyi, PCR for H. ducreyi and HPV, and cell atypia. Herpes simplex virus type 1 and donovaniasis were not tested due to high prevalence. |
| Downs et al., 2011 [48] | Tanzania | [Regional prevalence of S. haematobium, Northern villages, 10/337 (3.0%); Southern villages, 13/120 (10.8%), Regional prevalence of S. mansoni Northern villages, 41/337 (12.2%); HIV- infected 27/457 (5.9%), syphilis serology 33/457 (7.2%), trichomoniasis 15/457 (3.3%), candidiasis 22/457 (4.8%), bacterial vaginosis 33/457 (7.2%), HIV-Schistosomiasis coinfection 4/23 (17.4%)] | 457 | The study, conducted in partnership with a cervical cancer screening program in Tanzania, involved women aged 18-50 who were screened for FUS prevalence. Urine samples were collected and examined for schistosomal ova, Candida, Trichomonas vaginalis, and bacterial vaginosis. Abnormal cervical lesions were biopsied and examined for histopathological examination. Pregnant women were not included in the study. Venous blood was also collected and tested for syphilis using the rapid plasma reagin test and Treponema pallidum particle agglutination assay. |
| Downs et al., 2017 [49] | Tanzania | [Schistosomiasis-HIV-1-infected women, 20/45 (44.4%), Schistosomiasis-HIV-1-infected men, 8/28 (28.6%); HIV-uninfected women: Schistosome, 48/162 (29.6%); HIV-uninfected men: Schistosome, 39/103 (37.9%)] | 207 | The Kisesa observational HIV-1 cohort study has been surveyed and tested for HIV in Tanzania since 1994. The study includes seven villages near Lake Victoria and involves adults aged 15 and above. Data from archived dried blood spots collected during sero-surveys in 2007-2013 was used for the current study. The study confirmed positive results using the Vironostika Uni-Form Antigen/Antibody test and Enzygnost Anti-HIV1/2 Plus. |
| Shukla et al., 2023 [50] | South Africa | [STIs, 819/ 930 (88.1%); FGS, 210/933 (22.5%); Urinary S. haematobium ova, 256/840 (30.5%); HSV infection (serology), 286/900 (31.8%); Chlamydia, 218/827 (26.4%); HPV, 149/551 (27.0%); trichomoniasis, 163/ 839 (19.4%); gonorrhea, 102/827 (12.3%)] | 933 | A study involving sexually active, school-attending women aged 16-22 years, aged 10-12, was conducted from 2011 to 2013. The participants were tested for STIs and were diagnosed with FGS using a gynecological examination. Clinical manifestations of FGS were documented using photo-colposcopy. Herpes simplex virus type 2 (HSV-2) antibodies were detected in serum and cervical swabs. Syphilis was detected in thawed serum samples using Macro Vue test 110/112 and Immutrep for Treponema pallidum hemagglutination assay. The study used Chi-square or Fisher exact tests to evaluate the hypothesis and included age in multivariable logistic regression. The study used an α level of 0.05 for all statistical tests. |
| Leutscher et al.,2003 [51] | Madagascar | [Treponema pallidum infection; Andramahatana, 13/216 (16.3%), Androvakely, 22/80 (10.2%) ; HIV infection; Andramahatana, 2/216 (0.2%), Androvakely, 2/80 (2.5%); Schistosoma haematobium infection; Andramahatana, 67/216 (31.0%), Androvakely, 44/80 (55.0%)] | 438 | The study was conducted in three villages in Madagascar from 1998 to 2000. Men aged 15 to 49 were invited to a medical consultation, and urine samples were collected to estimate S hematobium egg count. Treponema pallidum antibodies were tested, and sera were screened for HIV antibodies using ELISA and Western blotting. |
| McCarthy et al.,1989 [52] | Sudan | [Northern soldiers: STD, 148/549 (28%); Southern soldiers: STD, 83/217 (38%); Northern soldiers: Schistosomiasis, 64/554 (12%); Southern soldiers: Schistosomiasis, 17/217 (8%) ] | 771 | Between 1987 and 1988, male Sudanese soldiers were surveyed about risk factors for hepatitis B and HIV-1 transmission, including sexual relations with prostitutes and previous STDs. Serologic markers were found, and stepwise multiple logistic regression analysis was employed. |
| [Downs et al., 2012](https://pubmed.ncbi.nlm.nih.gov/?term=Downs JA%5BAuthor%5D) [53] | Tanzania | [HIV-1, 21/345 (6%), Syphilis, 26/345 (8%), Gonorrhea, 1/345 (0.3%), Chlamydia, 15/345 (4%), Trichomoniasis, 4/345 (1%), Bacterial vaginosis, 19/345 (6%), Candidiasis, 15/345 (4%), HIV-Schistosome coinfection, 17/21 (81%), Schistosomiasis, 185/345 (54%)] | 345 | The study involved seven rural villages in western Tanzania with high rates of S. mansoni and HIV. It involved an oral questionnaire, gynecologic exam, and phlebotomy. Gynecologic examinations included wet preparations for Trichomonas vaginalis, Candida species, and bacterial vaginosis. Endocervical swabs were collected for Chlamydia trachomatis and Neisseria gonorrhoeae. Rapid tests were performed for schistosoma, C. trachomatis, and N. gonorrhoeae, and syphilis serology was performed using the Rapid Plasma Reagin test. |
| Walls et al.,2018 [54] | Zambia | [HIV-Schistosomiasis coinfection, (Male:599/2145 (28%), Female: 596/2145 (28%), Women; S. haematobium-HIV coinfection, 63 (30%), HIV infection; S. mansoni-HIV coinfection, 40 (19%), HIV infection, 167 (81%), Men:S. haematobium-HIV coinfection, 64 (33%), S. mansoni-HIV coinfection, 44 (22%), HIV infection, 152 (78%); Schistosomiasis, 1266/2145 (59%); Women: S. haematobium, 391 (26%), S. mansoni, 265 (18%); Men: S. haematobium, 492 (35%), S. mansoni, 197 (14%)] | 2145 | Between 1994 and 2012, heterosexual HIV discordant couples in Lusaka, Zambia, were enrolled in a longitudinal study. Genetically linked HIV infections were determined through PCR-amplified sequences. Plasma samples were tested for antibodies to schistosome soluble worm antigen preparation, stratified by gender, HIV status, and baseline characteristics. |
| Colombe et al.,2018 [55] | Tanzania | [HIV-1 seroconversion-Schistosomiasis, 23/43 (53.5%); HIV-1 seroconversion, 90/129 (69.8%); Schistosome infected, 26/43 (60.5%) ] | 172 | The study was part of the TAZAMA project, a community-based HIV-testing cohort in Kisesa, Tanzania, which has conducted sero-surveys on around 20,000 adults since 1994. The study identified seroconverters who became HIV-1-positive between September 2006 and February 2016, and used schistosome Circulating Anodic Antigen (CAA) in DBS collected during two consecutive sero-discordant sero-survey visits. The study confirmed HIV-1 infection using two different tests, the Uniform II Category III Ab test and the Enzygnost test. CD4 counts were measured using an automated BD FACS Calibur Machine. |
| Downs et al.,2017 [56] | Tanzania | [Women: HIV-1/Schistosomiasis coinfection, 20/45 (44.4%), Men:HIV-1/Schistosomiasis coinfection, 8/28 (28.6%); HIV- uninfected women; Schistosomiasis,48 (29.6%), HIV- uninfected men, 39 (37.9%)] | 674 | A case-control study was conducted within the Kisesa cohort, identifying HIV-1 seroconverters from two successive surveys. The seroconverters were selected randomly from all identified seroconverters. Dried blood spots were tested for HIV at the National Institute for Medical Research laboratory in Mwanza using the 4th-generation Vironostika Uni-Form Antigen/Antibody test. The study identified 73 HIV-1 seroconverters and 265 HIV- uninfected controls, with 4 controls for 55 cases and 3 controls for 9 cases. |
| Sanya et al.2015 [57] | Ugandan | [HIV infection, 17.3% (244/1412), S. mansoni infection, 57.2% (719/1257), HIV-S. mansoni, 116/719 (16.1%) ] | 1412 | A household survey was conducted in 26 fishing villages in Uganda, involving stool and urine samples for S. mansoni infection testing. The samples were analyzed in the field, using techniques like Kato-Katz analysis, urine-circulating cathodic antigen cassette test, and HIV antibody testing. The study aimed to identify and quantify S. mansoni infection. |
| Fontanet et al.,2000 [58] | Ethiopia | [Schistosome-HIV 10/52 (25.1 %); Schistosome infection 348/1187 (31.6%)] | 1239 | The Wonji-Shoa sugar estate in Ethiopia, established in 1953, conducts serological analysis of HIV-1, intestinal parasitic infections, and urine samples. The estate uses Vironistika ELISA for HIV-1 antibodies and western blotting for testing. Comparisons between groups are made using l, non-parametric tests, and t-tests for non-normal distribution variables. |
| Ndhlovu et al.,2007 [59] | Zimbabwe | [HIV infection prevalence: 156/544 (28.7%); S. Haematobium prevalence:72/216 (33.3%)] | 544 | The study involved 15-49-year-old women in Shamva district, Mupfure, who provided consent for HIV testing and gynecological examination, using urine samples and Chi-square statistics for comparison. |
| Midzi et al.,2017 [60] | Zimbabwe | [Among the 18 study participants with HIV-1 and urogenital schistosomiasis co-infection, 6 were ART naïve and 12 ART experienced; Schistosomiasis-HIV coinfection, 22/334 (6.6%); Schistosomiasis, 114/262 (43.5%)] | 1584 | The study compared HIV and S. haematobium co-infected men in Zimbabwe from April to October 2015. HIV-positive men aged 18 to 49 were recruited from the Ngundu Rural Health Centre. Screening for S. haematobum infection involved urine and stool samples, and the NucliSENSEasyQ HIV-1 v2.0 assay was used for HIV-1 RNA quantification. Sexually transmitted infection agents were assessed using the Nucliscense easyQ polymerase chain reaction (PCR) machine. |
| Mazigo et al.,2014 [61] | Tanzania | [S. mansoni was 854/1,785 (47.84%), HIV-1-S. mansoni coinfected, 50/125 (7.00%), HIV-1, 125/854 (14.6%) | 1785 | The study was conducted in Ilemela district, Mwanza region, Tanzania, between September 2012 and December 2012. Participants aged 21 to 55 years and living in the study villages for more than two years were eligible for enrollment. Human Immunodeficiency Virus-1 testing was conducted according to Tanzanian National HIV algorithms. Participants were counselled before and after testing. The study used Determine® and UNI-GOLD® for testing, and ELISA testing was performed for those with a positive test. A stool sample was collected and four Kato Katz thick smears were prepared for S. mansoni eggs. Comparisons of prevalence by demographic factors were tested for significance. |
| Kjetland et al.,2010 [62] | Zimbabwe | [HPV, 81 /236 (34%) and high-risk HPV in 54/236 (22%);HPV co-infection at follow-up was found in I 0/21 (48%) of the women with genital schistosomiasis, and 7/16 (44%) of women without genital schistoso¬ miasis; New HPV infection was found in 6/17 genital schistosorniasis-positive, versus 6/15 genital schistosorniasis-negative Persistent HPV subtype infection was found in 6/21 genital schistosomiasis-positive women and in 3/16 genital schistosomiasis-negative women. HIV was associated with HPV persistence (p = 0 047; Genital schistosomiasis, (270/55749% )) | 236 | A study in Zimbabwe involved 557 women aged 15-49, including those with HPV, and examined their health through various methods. The study focused on HPV detection using GP5+/6+ HPV PCR & EIA, and did not re-test for HIV in the 5-year study. Chi-square, Fisher's exact test, and odds ratio were used to compare prevalence. |
| Leutscher et al.,2005 [63] | Madagascar | [Baseline: Neisseria gonorrhoeae, 9/240 (3.8%); Chlamydia trachomatis ,12/240 (5%); Mycoplasma genitalium, 11/240 (4.6%); Trichomonas vaginalis, 15/240 (6.3%). Follow-up: Neisseria gonorrhoeae, 2/160 (1.3%); Chlamydia trachomatis ,1/160 (0.6%); Mycoplasma genitalium, 1/160 (0.6%); Trichomonas vaginalis, 7/160 (4.4%); HIV, 6/643 (0.9%); S. haematobium infection prevalence: at the baseline, 166/240 (69.2%), at follow-up, 29/160 (18.1%); Urine Egg detection, 160/240 (66.7%) &16/160 (10%) for baseline & follow-up respectively; Semen Egg detection, 67/240 (27.9%) &13/160 (8.1%)] | 240 | The study was a community-based study in the Ambilobe region, focusing on uro-genital schistosomiasis-associated morbidity. Participants aged 15-49 were included. The study measured Schistosoma egg excretion levels, cultured N. gonorrhoeae and Trichomonas vaginalis, diagnosed Chlamydia trachomatis and Mycoplasma genitalium, and tested HIV antibodies. Differences in proportions were compared. |
| Sturt et al.,2021 [64] | Zambia | Prevalence in Schistosomiasis negative sample: L. crispatus, 49/158 (31.0%), L. iners, 113/158 (71.5%), G. vaginalis, 115/158 (72.8%), A. vaginae, 112/158 (70.9%), T. vaginalis, 34/158 (21.5%), N. gonorrheae, 12/158 (7.6%), C. trachomatis, 13/158 (8.2%), M. genitalium, 7/158 (4.4%), C. albicans, 8/158 (5.1%), Schistosomiasis coinfections; L. crispatus, 6/16 (37.5%), L. iners, 11/16 (68.75%), G. vaginalis, 13/16 (81.3%), A. vaginae, 13/16 (81.3%), T. vaginalis, 8/16 (50.0%), C. trachomatis, , 2/16 (12.5%), C. albicans, 2/16 (12.5%) | 410 | The study analyzed the impact of an HIV-1 combination prevention package on bilharzia and HIV. It used CVL, vaginal, and cervical swab specimens for PCR detection of Schistosoma, characterization of microbiota and STI, and urine for detection of CAA and S. haematobium eggs. The study quantified Lactobacillus crispatus as a key marker of vaginal health, characterized markers of a "nonoptimal" cervicovaginal microbiota, and quantified STI. |
| Yirenya-Tawiah et al., 2013 [65] | Ghana | [schistosomiasis (FGS), 42/395 (10.6%), Candida albicans, 59/377 (15.6%), Trichomonas vaginalis, 6/392 (1.5%), Bacteria vaginosis, 53/392 (13.5%), HIV, 29/402 (7.2%), Schistosomiasis-HIV coinfection, 4/41 (9.8%) | 402 | This 2005 cross-sectional study examined female genital schistosomiasis in 16 communities in six local government districts. Women who consented were examined medically and screened for demographics, urogenital symptoms, and obstetric and gynaecological history. Cervical biopsy was used for genital schistosomiasis detection, while high vaginal swabs and venous blood were collected for genital infections and HIV. The study identified C. albicans, T vaginalis, and B. vaginosis, and diagnosed genital schistosomiasis using compressed biopsy techniques. A single HIV test was performed, and Chi square and Fisher exact tests were used to assess the association between variables. |
| Kallestrup et al.,2005 [66] | Zimbabwe | [HIV-1 prevalence (407/1545 (26.3%); Schistosoma haematobium prevalence, 426/1545 (27.6%); S. mansoni prevalence, 121/1545 (7.8%); S. haematobium and S. mansoni coinfection prevalence, 123/1545 (8%) CAA results: Schistosoma haematobium prevalence, 206/356 (57.9%); S. mansoni prevalence, 24/356 (6.7%); S. haematobium and S. mansoni prevalence, 37/356 (10.4%)] | 1545 | This 2005 cross-sectional study examined female genital schistosomiasis in 16 communities in six local government districts. Women who consented were examined medically and screened for demographics, urogenital symptoms, and obstetric and gynaecological history. Cervical biopsy was used for genital schistosomiasis detection, while high vaginal swabs and venous blood were collected for genital infections and HIV. The study identified C. albicans, T vaginalis, and B. vaginosis, and diagnosed genital schistosomiasis using compressed biopsy techniques. A single HIV test was performed, and Chi square and Fisher exact tests were used to assess the association between variables. |
| Kutz et al., 2023 [67] | Madagascar | HPV, 129/302 (42.7%); Schistosomiasis (FGS), 189/302 (62.6%); Schistosomiasis-HPV coinfection, 80/302 (36.5%) | 302 | A cross-sectional study was conducted at three PHCCs in Marovoay, Madagascar, between December 2020 and February 2021. Participants were aged 18-49, fluent in French or Malagasy, and provided voluntary written informed consent. HPV DNA was detected using E7 PCR bead-based multiplex genotyping assay. |
| Yang et al.,2018 [68] | southwestern China | [HIV+/SjAb− (n=32), , HIV+/SjAb+ (n =16), and HIV−/SjAb− (n =29); HIV−/SjAb+ (n =13) ] | 90 | The study involved 90 Yi individuals aged over 3 years without metabolic or autoimmune diseases in Puge County, Liangshan Yi Prefecture, China. They were screened for CD4+ and CD8+ T lymphocyte counts and cytokine levels using a blood sample. Participants were also tested for HIV antibody and S. japonicum antibodies. The samples were sent to the local CDC laboratory for examination. Kruskal-Wallis rank tests were used to compare median T lymphocyte counts and cytokine levels. |
| Njoku, 2014 [69] | Nigeria | [HIV prevalence was 27/396 (6.8%); S. haematobium eggs in urine of apparently healthy populations was 265/1007 (26.3%)] | 1007 | Jos, Nigeria's capital, is conducting HIV testing on 197 APH, urinary schistosomiasis positive individuals, and HIV/urinary schistosomiasis co-infected individuals through rapid ELISA technique. |
| Prodger et al.,2015 [70] | Ugandan | [S. Mansoni-HSV-2 Seropositive, 4/12 (33.3%), HSV-2, 1/12 (8.4%)] | 34 | A study was conducted on 18-49-year-olds from the Uganda Virus Research Institute-Inter-national AIDS Vaccine Initiative (UVRI-IAVI) Voluntary HIV Counselling and Testing Clinic in Kasenyi, Uganda. Participants were screened for HIV using the Uganda National Algorithm, including two rapid tests and a third rapid test. Urine samples were collected for three consecutive days before, during, and after surgery. Screening for S. mansoni was performed using the Kato-Katz method, while urine-CCA was detected using the Rapid Medical Diagnostics Schistosomiasis Test. The study used the Friedman chi-square test and the Wilcoxon rank test. |

**References**

1. Mayaud P, Changalucha J, Grosskurth H, Ka-Gina G, Rugemalila J, Nduba J, Newell J, Hayes R, Mabey D. The value of urine specimens in screening for male urethritis and its microbial aetiologies in Tanzania. Sexually Transmitted Infections. 1992 Dec 1;68(6):361-5.
2. Ansart S, Perez L, Vergely O, Danis M, Bricaire F, Caumes E. Illnesses in travelers returning from the tropics: a prospective study of 622 patients. Journal of Travel Medicine. 2005 Nov 1;12(6):312-8.
3. Kjetland EF, Kurewa EN, Ndhlovu PD, Midzi N, Gwanzura L, Mason PR, Gomo E, Sandvik L, Mduluza T, Friis H, Gundersen SG. Female genital schistosomiasis–a differential diagnosis to sexually transmitted disease: genital itch and vaginal discharge as indicators of genital Schistosoma haematobium morbidity in a cross‐sectional study in endemic rural Zimbabwe. Tropical Medicine & International Health. 2008 Dec;13(12):1509-17.
4. Hegertun IE, Sulheim Gundersen KM, Kleppa E, Zulu SG, Gundersen SG, Taylor M, Kvalsvig JD, Kjetland EF. S. haematobium as a common cause of genital morbidity in girls: a cross-sectional study of children in South Africa. PLoS neglected tropical diseases. 2013 Mar 21;7(3):e2104.
5. Downs JA, Kabangila R, Verweij JJ, Jaka H, Peck RN, Kalluvya SE, Changalucha JM, Johnson WD, van Lieshout L, Fitzgerald DW. Detectable urogenital schistosome DNA and cervical abnormalities 6 months after single‐dose praziquantel in women with S chistosoma haematobium infection. Tropical Medicine & International Health. 2013 Sep;18(9):1090-6.
6. Galappaththi-Arachchige HN, Amlie Hegertun IE, Holmen S, Qvigstad E, Kleppa E, Sebitloane M, Ndhlovu PD, Vennervald BJ, Gundersen SG, Taylor M, Kjetland EF. Association of urogenital symptoms with history of water contact in young women in areas endemic for S. haematobium. a cross-sectional study in rural South Africa. International journal of environmental research and public health. 2016 Nov;13(11):1135.
7. Galappaththi-Arachchige HN, Zulu SG, Kleppa E, Lillebo K, Qvigstad E, Ndhlovu P, Vennervald BJ, Gundersen SG, Kjetland EF, Taylor M. Reproductive health problems in rural South African young women: risk behaviour and risk factors. Reproductive health. 2018 Dec;15:1-0.
8. Yegorov S, Galiwango RM, Good SV, Mpendo J, Tannich E, Boggild AK, Kiwanuka N, Bagaya BS, Kaul R. Schistosoma mansoni infection and socio-behavioural predictors of HIV risk: a cross-sectional study in women from Uganda. BMC infectious diseases. 2018 Dec;18:1-9.
9. Gadoth A, Mvumbi G, Hoff NA, Musene K, Mukadi P, Ashbaugh HR, Doshi RH, Javanbakht M, Gorbach P, Okitolonda-Wemakoy E, Klausner JD. Urogenital schistosomiasis and sexually transmitted coinfections among pregnant women in a schistosome-endemic region of the Democratic Republic of Congo. The American journal of tropical medicine and hygiene. 2019 Oct;101(4):828.
10. Kjetland EF, Ndhlovu PD, Gomo E, Mduluza T, Midzi N, Gwanzura L, Mason PR, Sandvik L, Friis H, Gundersen SG. Association between genital schistosomiasis and HIV in rural Zimbabwean women. Aids. 2006 Feb 28;20(4):593-600.
11. Downs JA, Mguta C, Kaatano GM, Mitchell KB, Bang H, Simplice H, Kalluvya SE, Changalucha JM, Johnson Jr WD, Fitzgerald DW. Urogenital schistosomiasis in women of reproductive age in Tanzania's Lake Victoria region. The American journal of tropical medicine and hygiene. 2011 Mar 3;84(3):364.
12. Downs JA, Dupnik KM, van Dam GJ, Urassa M, Lutonja P, Kornelis D, de Dood CJ, Hoekstra P, Kanjala C, Isingo R, Peck RN. Effects of schistosomiasis on susceptibility to HIV-1 infection and HIV-1 viral load at HIV-1 seroconversion: a nested case-control study. PLoS neglected tropical diseases. 2017 Sep 25;11(9):e0005968.
13. Shukla JD, Kleppa E, Holmen S, Ndhlovu PD, Mtshali A, Sebitloane M, Vennervald BJ, Gundersen SG, Taylor M, Kjetland EF. The association between female genital schistosomiasis and other infections of the lower genital tract in adolescent girls and young women: A cross-sectional study in South Africa. Journal of Lower Genital Tract Disease. 2023 Jul 1;27(3):291-6.
14. Leutscher PD, Behets F, Rousset D, Ramarokoto CE, Siddiqi O, Ravaoalimalala EV, Christensen NO, Migliani R. Sexual behavior and sexually transmitted infections in men living in rural Madagascar: implications for HIV transmission. Sexually transmitted diseases. 2003 Mar 1;30(3):262-5.
15. McCarthy MC, Hyams KC, El-Tigani El-Hag A, El-Dabi MA, El-Sadig El-Tayeb M, Khalid IO, George JF, Constantine NT, Woody JN. HIV-1 and hepatitis B transmission in Sudan. Aids. 1989 Nov 1;3(11):725-9.
16. Downs JA, van Dam GJ, Changalucha JM, Corstjens PL, Peck RN, de Dood CJ, Bang H, Andreasen A, Kalluvya SE, van Lieshout L, Johnson Jr WD. Association of Schistosomiasis and HIV infection in Tanzania. The American journal of tropical medicine and hygiene. 2012 Nov 11;87(5):868.
17. Wall KM, Kilembe W, Vwalika B, Dinh C, Livingston P, Lee Y-M, et al. (2018) Schistosomiasis is associated with incident HIV transmission and death in Zambia. PLoS Negl Trop Dis 12(12): e0006902.
18. Colombe S, Corstjens PL, de Dood CJ, Miyaye D, Magawa RG, Mngara J, Kalluvya SE, Van Lieshout L, Van Dam GJ, Downs JA. HIV-1 viral loads are not elevated in individuals co-infected with Schistosoma spp. after adjustment for duration of HIV-1 infection. Frontiers in Immunology. 2018 Sep 6;9:2005.
19. Downs JA, de Dood CJ, Dee HE, McGeehan M, Khan H, Marenga A, Adel PE, Faustine E, Issarow B, Kisanga EF, Kisigo GA. Schistosomiasis and human immunodeficiency virus in men in Tanzania. The American journal of tropical medicine and hygiene. 2017 Apr 4;96(4):856.
20. Sanya RE, Muhangi L, Nampijja M, Nannozi V, Nakawungu PK, Abayo E, Webb EL, Elliott AM, LaVIISWA Study Team, Nampijja M, Sanya R. Schistosoma mansoni and HIV infection in a Ugandan population with high HIV and helminth prevalence. Tropical medicine & international health. 2015 Sep;20(9):1201-8.
21. Fontanet AL, Woldemichael T, Sahlu T, Van Dam GJ, Messele T, Rinke de Wit T, Masho W, Yeneneh H, Coutinho RA, Van Lieshout L. Epidemiology of HIV and Schistosoma mansoni infections among sugar-estate residents in Ethiopia. Annals of Tropical Medicine & Parasitology. 2000 Mar 1;94(2):145-55.
22. Ndhlovu PD, Mduluza T, Kjetland EF, Midzi N, Nyanga L, Gundersen SG, Friis H, Gomo E. Prevalence of urinary schistosomiasis and HIV in females living in a rural community of Zimbabwe: does age matter?. Transactions of the Royal Society of Tropical Medicine and Hygiene. 2007 May 1;101(5):433-8.
23. Midzi N, Mduluza T, Mudenge B, Foldager L, Leutscher PD. Decrease in seminal HIV-1 RNA load after praziquantel treatment of urogenital schistosomiasis coinfection in HIV-positive men—an observational study. InOpen Forum Infectious Diseases 2017 (Vol. 4, No. 4, p. ofx199). US: Oxford University Press.
24. Mazigo HD, Dunne DW, Wilson S, Kinung’hi SM, de Moira AP, Jones FM, Morona D, Nuwaha F. Co-infection with Schistosoma mansoni and Human Immunodeficiency Virus-1 (HIV-1) among residents of fishing villages of north-western Tanzania. Parasites & vectors. 2014 Dec;7:1-9.
25. Kjetland EF, Ndhlovu PD, Mduluza T, Deschoolmeester V, Midzi N, Gomo E, Gwanzura L, Mason PR, Vermorken JB, Friis H, Gundersen SG. The effects of genital Schistosoma haematobium on human papillomavirus and the development of cervical neoplasia after five years in a Zimbabwean population-The impact of Schistosoma haematobium on persistent HPV in rural Zimbabwean women at high-risk was investigated. European journal of gynaecological oncology. 2010 Jan 1;31(2):169.
26. Leutscher PD, Pedersen M, Raharisolo C, Jensen JS, Hoffmann S, Lisse I, Ostrowski SR, Reimert CM, Mauclere P, Ullum H. Increased Prevalence of Leukocytes and Elevated Cytokine Levels in Semen from Schistosoma haematobium—Infected Individuals. Journal of Infectious Diseases. 2005 May 15;191(10):1639-47.
27. Sturt AS, Webb EL, Himschoot L, Phiri CR, Mapani J, Mudenda M, Kjetland EF, Mweene T, Levecke B, van Dam GJ, Corstjens PL. Association of female genital Schistosomiasis with the cervicovaginal microbiota and sexually transmitted infections in Zambian women. InOpen Forum Infectious Diseases 2021 Sep 1 (Vol. 8, No. 9, p. ofab438). US: Oxford University Press.
28. Yirenya-Tawiah DR, Amoah CM, Apea-Kubi KA, Dade M, Lomo G, Mensah D, Akyeh L, Bosompem KM. Female genital schistosomiasis, genital tract infections and HIV co-infection in the Volta basin of Ghana. International Journal of TROPICAL DISEASE & Health. 2013 Mar 17;3(2):94-103.
29. Kallestrup P, Zinyama R, Gomo E, Butterworth AE, van Dam GJ, Erikstrup C, Ullum H. Schistosomiasis and HIV-1 infection in rural Zimbabwe: implications of coinfection for excretion of eggs. The Journal of infectious diseases. 2005 Apr 15;191(8):1311-20.
30. Kutz JM, Rausche P, Rasamoelina T, Ratefiarisoa S, Razafindrakoto R, Klein P, Jaeger A, Rakotomalala RS, Rakotomalala Z, Randrianasolo BS, McKay-Chopin S. Female genital schistosomiasis, human papilloma virus infection, and cervical cancer in rural Madagascar: a cross sectional study. Infectious Diseases of Poverty. 2023 Sep 25;12(1):89.
31. Yang Y, Xiao PL, Yang Y, Gao JC, Shi Y, Cheng WT, Chen Y, Song XX, Jiang QW, Zhou YB. Immune dysfunction and coinfection with human immunodeficiency virus and schistosoma japonicum in yi people. Journal of Immunology Research. 2018 Jul 2;2018.
32. Njoku JC. Epidemiological Study of Urogenital Schistosomiasis in Apparently Health and HIV-Infected Females in Jos, Plateau State, Nigeria (Doctoral dissertation).
33. Prodger JL, Ssemaganda A, Ssetaala A, Kitandwe PK, Muyanja E, Mpendo J, Nanvubya A, Wambuzi M, Nielsen L, Kiwanuka N, Kaul R. Schistosoma mansoni infection in Ugandan men is associated with increased abundance and function of HIV target cells in blood, but not the foreskin: a cross-sectional study. PLoS neglected tropical diseases. 2015 Sep 3;9(9):e0004067.
